# The Influence of Dietary Fatty Acids on Immune Responses

**DOI:** 10.3390/nu11122990

**Published:** 2019-12-06

**Authors:** Urszula Radzikowska, Arturo O. Rinaldi, Zeynep Çelebi Sözener, Dilara Karaguzel, Marzena Wojcik, Katarzyna Cypryk, Mübeccel Akdis, Cezmi A. Akdis, Milena Sokolowska

**Affiliations:** 1 Swiss Institute of Allergy and Asthma Research (SIAF), University of Zurich, 7265 Davos Wolfgang, Switzerland; urszula.radzikowska@siaf.uzh.ch (U.R.);; 2 Christine Kühne-Center for Allergy Research and Education, 7265 Davos Wolfgang, Switzerland; 3 Department of Regenerative Medicine and Immune Regulation, Medical University of Bialystok, 15-269 Bialystok, Poland; 4 Department of Chest Disease, Division of Allergy and Clinical Immunology, Ankara University School of Medicine, 06100 Ankara, Turkey; 5 Department of Biology, Faculty of Science, Hacettepe University, 06800 Ankara, Turkey; 6 Department of Structural Biology, Medical University of Lodz, 90-752 Lodz, Poland; 7 Department of Internal Medicine and Diabetology, Medical University of Lodz, 90-549 Lodz, Poland

**Keywords:** innate lymphoid cell, T cell, B cell, epithelium, macrophage, neutrophil, allergy, asthma, autoimmune disease, diabetes

## Abstract

Diet-derived fatty acids (FAs) are essential sources of energy and fundamental structural components of cells. They also play important roles in the modulation of immune responses in health and disease. Saturated and unsaturated FAs influence the effector and regulatory functions of innate and adaptive immune cells by changing membrane composition and fluidity and by acting through specific receptors. Impaired balance of saturated/unsaturated FAs, as well as *n*-6/*n*-3 polyunsaturated FAs has significant consequences on immune system homeostasis, contributing to the development of many allergic, autoimmune, and metabolic diseases. In this paper, we discuss up-to-date knowledge and the clinical relevance of the influence of dietary FAs on the biology, homeostasis, and functions of epithelial cells, macrophages, dendritic cells, neutrophils, innate lymphoid cells, T cells and B cells. Additionally, we review the effects of dietary FAs on the pathogenesis of many diseases, including asthma, allergic rhinitis, food allergy, atopic dermatitis, rheumatoid arthritis, multiple sclerosis as well as type 1 and 2 diabetes.

## 1. Introduction

**Figure 1:**
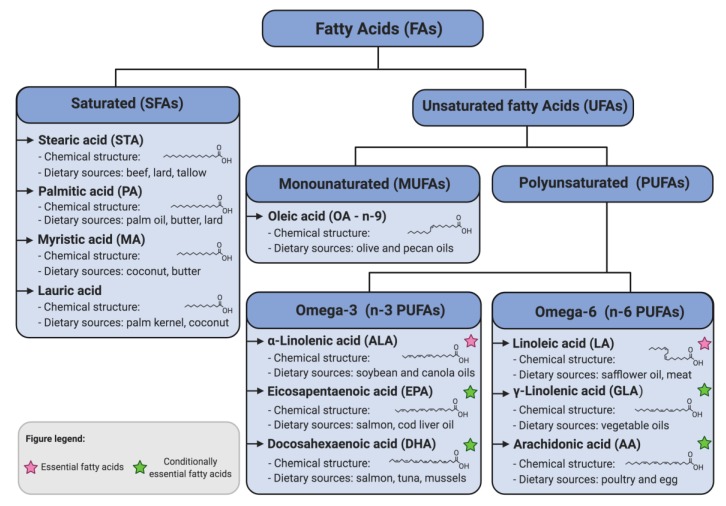
Classification, chemical structure, and primary dietary sources of fatty acids (FAs). The figure includes only dietary FAs discussed in this paper. For full classification of FAs, see [13]. For details, see the text.

Fatty acids (FAs) are carboxylic acids containing a different number of carbons and double bonds [1]. Based on the chemical structure and subsequent biochemical properties, FAs can be divided into two main groups. The first group is comprised of saturated FAs (SFAs) such as palmitic acid (PA; C16:0), lauric acid (C12:0), myristic acid (MA; C14:0), and stearic acid (STA; C18:0). These FAs do not contain any double bonds in their carbon backbone. The second group consists of unsaturated FAs (UFAs) containing one (monounsaturated FAs; MUFAs) or more (polyunsaturated FAs; PUFAs) double bonds in their structure. MUFAs include FAs such as *n*-9 oleic acid (OA; C18:1) and others, discussed elsewhere [2]. The PUFA family includes α-Linolenic acid (ALA; C18:3), linoleic acid (LA; C18:2) and their long-chain derivatives (Figure 1). The main sources of SFAs are meats, dairy products, palm, and coconut oils, whereas the primary sources of UFAs are vegetable oils, nuts, and fish (Figure 1). FAs in humans are ingested with foods and they are synthesized by cells. However, mammals lack the enzymes to introduce double bonds at carbon atoms beyond C-9 in the fatty acid chain. Hence, humans cannot synthesize LA, also called linoleate, an *n*-6 PUFA, derived from meat, or ALA, also called linolenate, an *n*-3 PUFA, derived from vegetable oils, such as soybean or canola oil [3,4]. Therefore, these two FAs are considered essential and must be supplied by the diet to be the starting point for the synthesis of a variety of other UFAs. Long-chain derivatives of those essential PUFAs, such as *n*-6 PUFAs γ-linolenic acid (GLA; C18:3) and arachidonic acid (AA; C20:4) as well as *n*-3 PUFAs docosahexaenoic acid (DHA; C22:6) and eicosapentaenoic acid (EPA; C20:5) are considered highly necessary for various physiological functions at every stage of human life [5]. They are synthesized within the body, but they also should be delivered with foods. Thus, they are sometimes called conditionally essential FAs [3,6,7] (Figure 1). 

FAs play several essential roles in the homeostasis and structure of the cell and the whole human body [8]. First of all, they are the main components of all biological membranes built into sphingolipids, phospholipids, glycolipids, and lipoproteins. Secondly, they are the source of energy stored in the triacylglycerols. Finally, various metabolites of FAs serve as essential intracellular and extracellular lipid mediators and hormones [8,9,10,11,12]. Therefore, FAs have nearly infinite possibilities to modulate immune functions of the cell by influencing its structure, metabolism, and function, acting through surface proteins (G-protein-coupled receptors; GPRs), intranuclear receptors or membrane transporters [8].

Immunomodulatory properties of dietary FAs during systemic inflammation have been investigated in both healthy and diseased people [14,15,16,17,18,19,20]. The increased consumption of SFAs from processed foods is associated with an increased risk of atherosclerosis, coronary heart disease, obesity, and metabolic syndrome, which has been extensively reviewed elsewhere [21,22,23,24]. The consumption of UFAs constitutes either protection or risk of development of many immune-related and metabolic diseases depending on the *n*-6/*n*-3 PUFAs balance, diet diversity, underlying genetics, and other environmental factors including microbiome [25,26]. In this work, we comprehensively describe the up-to-date view on the immunomodulatory effects of dietary FAs on the cells of innate and adaptive immunity, together with their involvement in the pathogenesis of allergic, autoimmune, and some metabolic diseases.

## 2. Dietary Fatty Acids and Innate Immune Responses

### 2.1. Epithelium

The epithelium constitutes a biochemical and physical mucosal barrier between the body’s internal milieu and the environment [27]. Epithelial cells (ECs) in the lungs, gut, and the skin are the first line of protection from potentially harmful factors, present in the external environment. Mucosal barriers are built from immunologically active cells, tightly attached by tight junction proteins (TJs). They also produce mucus and control tissue clearance by cilia movement [27,28,29]. ECs express several pattern recognition receptors (PRRs) and, in response to pathogen-associated molecular patterns (PAMPs) and danger-associated molecular patterns (DAMPs), secrete a broad panel of potent pro- and anti-inflammatory cytokines, chemokines, and lipid mediators [27,28,30]. By direct and indirect interactions with other innate (macrophages, neutrophils, dendritic cells, innate lymphoid cells) and adaptive (T and B cells) immune cells, ECs actively shape local and systemic immune responses [27,28,29]. 

Intestinal epithelial cells (IECs) ensure gut homeostasis by a careful balance between the absorption of crucial nutrition factors, such as FAs, and blocking the infiltration of harmful molecules, such as pathogenic bacteria [27,29]. The effects of *n*-3 and *n*-6 PUFAs on the physical and immunological function of the intestinal barrier have been broadly investigated in the in vitro models. The stimulation of the T84 intestinal epithelial cell line with DHA and EPA restores previous impairment by tumor necrosis factor-alpha (TNF-α) and interferon-gamma (IFN-γ) membrane permeability, increases Trans Epithelial Electrical Resistance (TEER), and reconstructs the correct morphology and distribution of TJs by modifying the lipid environment in TJ membrane microdomains [31]. Not only *n*-3 (EPA, DHA) but also *n*-6 (dihomo-γ-linolenic acid and AA) PUFAs improve intestinal barrier integrity after interleukin-4 (IL-4)-mediated IEC disruption [32]. Similarly, in the presence of inflammatory mediators such as IL-1β, TNF-α, IFN-γ, and lipopolysaccharide (LPS), DHA-dependent improvement of the barrier function is observed [33]. The anti-inflammatory effects of ALA, DHA, EPA, and GLA were also investigated in the IL-1β-mediated inflammation model in the Caco-2 cell line [34]. All these PUFAs decrease the secretion of inflammatory cytokines such as IL-6 and/or IL-8 [34]. This effect in DHA- and EPA-stimulated cells is mediated by the nuclear receptor, peroxisome proliferator-activated receptor-gamma (PPARγ) [34]. Additionally, GPR120 is an important transmembrane receptor for both *n*-3 and *n*-6 PUFAs [35,36]. In the Caco-2 cells, EPA, DHA, and AA acting through GPR120, but not GPR40, activate accumulation of cytosolic calcium, induce the extracellular signal-regulated kinase 1/2 (ERK1/2) mitogen-activated protein kinase (MAPK) signaling pathway and decrease in IL-1β-induced NF-κB activation [36]. The protective effects of *n*-3 PUFAs in the gut are repeatedly confirmed in the in vivo set up. In the mouse model of intestinal anaphylactic response to the cow’s milk, low-dose *n*-3 PUFA (fish oil) supplementation decreases electrophysiological parameters such as short-current circuit and tissue conductance, followed by the improved morphological composition of the jejunal tissue [37]. In the IL-10-deficient mouse model of chronic colitis, supplementation with DHA significantly reduces the inflammatory score, prevents body weight loss, and decreases the production of pro-inflammatory cytokines (IL-17, TNF-α, and IFN-γ) [38]. Additionally, DHA treatment in this model improves the barrier function of the gut, demonstrated as decreased permeability, increased TEER, and enhancement of TJ expression (zonula occludens 1 and occludin) [38]. Similarly, animals suffering from severe combined immunodeficiency, fed with the *n*-3 PUFAs rich diet before and after colitis induction, show decreased intestinal pathology scores, increased zonula occludens 1 expression in the IECs, and decreased the production of pro-inflammatory cytokines (IL-12, TNF-α, IL-1β) [39]. The reduction of oxidative stress is another mechanism of the protective effects of *n*-3 PUFAs (ALA) in the intestine and is represented as decreased urinary 8-isoprostanes production, normalized colon glutathione levels and decreased inducible nitric oxide synthase expression in a model of colitis in rats [40]. 

The airway epithelium (AECs) is the first line of defense in the respiratory system. Similarly, to the impact observed in the gut, *n*-3 PUFAs have anti-inflammatory effects in the lungs. DHA supplementation decreases IL-6 and IFN-γ-induced protein 10 kDa (IP-10) secretion from AECs after rhinovirus infection [41], reflecting the protective effects of *n*-3 PUFAs in the airways in the presence of underlying inflammatory condition. The influence of DHA was also investigated in primary bronchial epithelial cells, the bronchial epithelial cell line (BEAS-2B) and in mice exposed to the dust extract (DE) [42]. Stimulation with DHA restores healing capacity in the DE-treated epithelium [42]. In contrast, Calu-3 cell lines treated with DHA alone show increased permeability and mucus production, as well as decreased TEER [43]. Similarly, *n*-6 PUFAs such as LA, GLA, and AA have negative effects on barrier function [43]. Additionally, AA treatment increases cytotoxicity (lactate dehydrogenase release) and the secretion of IL-6 in Calu-3 cells [43,44]. Those results indicate that the effects of PUFAs on AECs can be pro- or anti-inflammatory, and *n*-6/*n*-3 PUFAs content in the cellular membrane is crucial for the beneficial effects [41,43,44]. Furthermore, a Western diet causing an imbalance in the FA intake (*n*-6 PUFA levels are higher than *n*-3 PUFAs) is associated with obesity-related asthma [45,46]. An enhanced *n*-6/*n*-3 PUFA ratio increases the risk of uncontrolled asthma, characterized by severe clinical outcomes and frequent exacerbations [47]. The stimulation of BEAS-2B with *n*-6 PUFAs (AA), combined with rhinovirus infection, substantially induces the expression of pro-inflammatory cytokines IL-6 and IL-8 [48]. This effect is dependent on c-Jun N-terminal kinase (JNK) and p38 MAPK signaling [48]. It all suggests a possible link between increased dietary *n*-6 PUFA intake and poor asthma control. 

**Figure 2:**
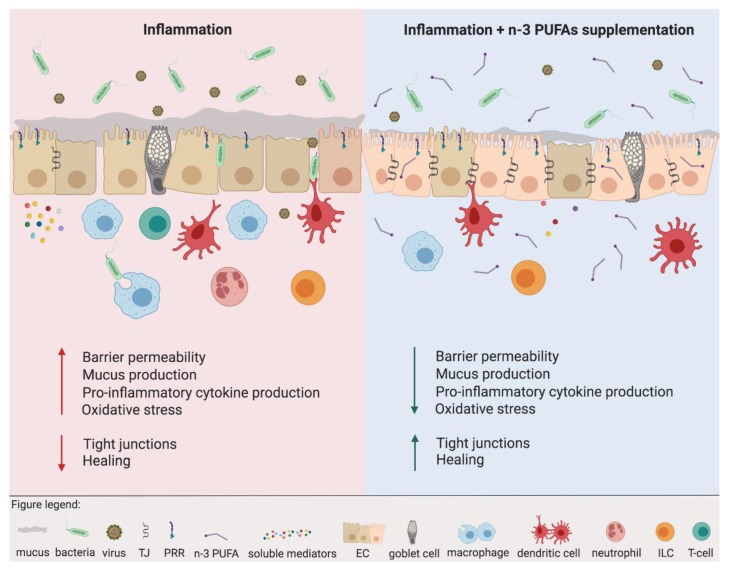
Protective effects of dietary fatty acids on the epithelium. For details, see the text. ↓—downregulation or decrease; ↑—upregulation or increase; TJ—Tight Junction; PRR—Pattern Recognition Receptor; *n*-3 PUFA—*n*-3 Polyunsaturated Fatty Acid; EC—Epithelial Cell; ILC—Innate Immune Cell

In summary, the beneficial influence of *n*-3 PUFAs on epithelial cells comes from the activation of nuclear and transmembrane receptors, as well as from restoration of the *n*-6/*n*-3 PUFAs content of the cellular membranes. In the presence of underlying inflammation, *n*-3 PUFAs can restore impaired barrier function and reduce the production of pro-inflammatory mediators (Figure 2, Table 1; Table 2). However, the majority of the discussed data come from in vitro studies and animal models. Less is known about the direct clinical relevance of the observed results. Several clinical trials investigated the importance of *n*-3 PUFA supplementation in epithelium-related diseases in the gut and lung such as inflammatory bowel diseases and asthma. Unfortunately, those studies are rather inconclusive. Some current data suggest a positive, whereas others demonstrate no or even a negative effect of *n*-3 PUFAs in patients. We discuss the effects of dietary FAs in the course of asthma in Section 4.1.1, whereas the role of *n*-3 PUFAs in inflammatory bowel disease (IBD) is reviewed elsewhere [49,50,51,52]. 

### 2.2. Macrophages

Macrophages are essential tissue-resident members of the innate immune system. Due to the significant functional and phenotypic heterogeneity, they are involved not only in the clearance of pathogens, phagocytosis, and anti—gen presentation but also in tissue repair [138,139]. Activated macrophages undergo a differentiation process in response to various microenvironmental stimulants, generating M1-like, M2-like, or other less-defined phenotypes [140]. M1-like macrophages are responsible for secreting pro-inflammatory cytokines and lipid mediators and are involved in tissue degradation and T cell activation. M2 macrophages exert different functions, such as contribution to tissue repair and the secretion of anti-inflammatory cytokines and lipid mediators [141,142,143].

Dietary FAs are potent modulators of macrophage functions. It has been shown that some PUFAs, especially DHA via the GPR120 receptor, drive M2-like differentiation with increased phagocytic activity and induce IL-10 release [72,73]. In addition, DHA-stimulated M2-like polarization in macrophage cell lines (human U937 and murine RAW264.7) is regulated by p38 MAPK and mediated via PPARγ [70,71]. DHA, via GPR120, can also activate cytosolic phospholipase A2 (cPLA_2_), leading to the release of AA from the cellular membranes, activation of cyclooxygenase 2 (COX-2), and release of prostaglandin E_2_ (PGE_2_) in RAW264.7 and in human primary monocyte-derived macrophages. It has been shown that this effect is anti-inflammatory through the PGE_2_-mediated signaling on EP4 [74]. DHA has higher anti-inflammatory potency than EPA and, intriguingly, combined DHA and EPA treatment shows stronger immunomodulatory efficacy as compared to any of these *n*-3 PUFAs alone [144,145]. In contrast to DHA, SFAs, such as PA, induces the secretion of pro-inflammatory IL-18, and TNF-α [96].

Adipose tissue macrophages are immunologically active cells, actively participating in the pathophysiology of obesity. Interestingly, dietary FAs significantly influence their homeostasis. Oh et al. reported, that diet rich in DHA and EPA (fish oil) not only reduces the frequency of macrophages in the adipose tissue but also induces a shift to M2 anti-inflammatory phenotype, represented by the increased expression of IL-10, arginase, YM-1, Clec7a and MMR genes [72]. Additionally, it has been shown that both *n*-3 (DHA) and *n*-6 (AA) PUFAs increase the phagocytic and microbicidal activity of RAW264.7 cells, suggesting a beneficial role of PUFA supplementation in immunocompromised patients with chronic infections [106]. These data are confirmed in vivo in a murine model of *Streptococcus pneumoniae* infection [107]. Saini et al. demonstrated that the *n*-3 PUFA-rich diet increases phagocytosis and decreases the apoptosis of alveolar macrophages [107]. In addition, dietary FAs have been shown to act on Toll-like receptors (TLRs). Interestingly, DHA and EPA can modulate TLR4-mediated immune signaling and inhibit LPS-mediated COX-2 activation and subsequent prostaglandin release [53,54]. On the other hand, TLR4 on macrophages acts as the main receptor for SFAs, such as PA, which leads to an inflammatory response through the NF-κB pathway [95]. Anti-inflammatory properties of DHA and EPA also include the inhibition of the NOD-like receptor protein 3 (NLRP3) inflammasome activation in macrophages [108,109]. This effect is mediated in vitro and in vivo by GPR120 and GPR40 receptors, their downstream protein β-arrestin-2 [109], and autophagy induction [110]. Interestingly, pro-inflammatory properties of SFAs (PA) are confirmed in the inflammasome-related inflammation. Snodgrass et al. demonstrated, that PA-dependent TLR2 activation leads to a NLRP3 inflammasome activation and an induction of IL-1β secretion in the THP-1 cell line [97], which points out the importance of the right balance between the SFAs and UFAs.

**Figure 3:**
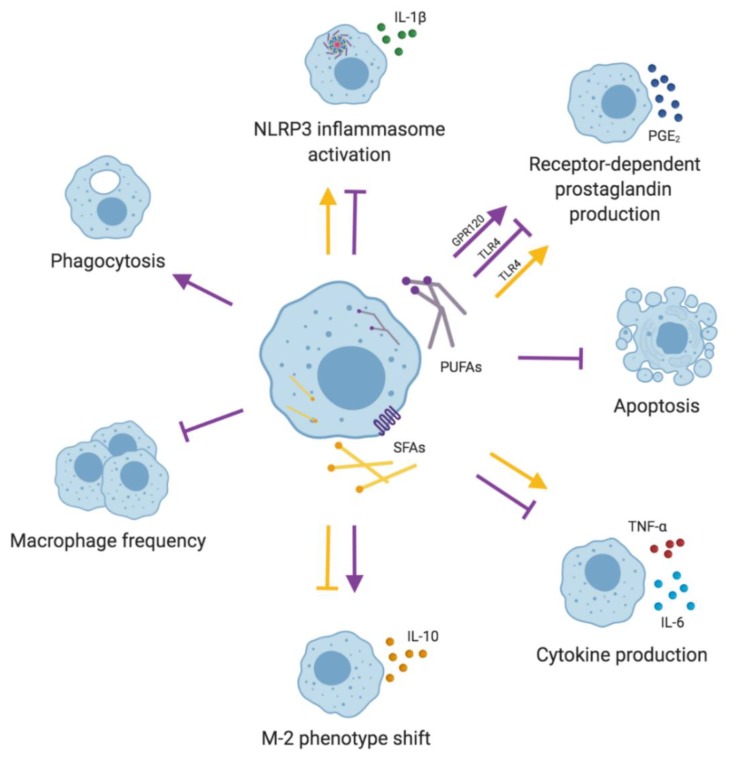
Anti-inflammatory and pro-inflammatory effects of dietary fatty acids on macrophages. For details, see the text. ⊥—inhibition; ↑—activation; PUFAs—Polyunsaturated Fatty Acids; SFAs—Saturated Fatty Acids; NLRP3—NOD-like Receptor Protein 3; IL-1β—Interleukin-1 Beta; PGE_2_—Prostaglandin E2; GPR120—G-protein Coupled Receptor 120; TLR4—Toll-like Receptor 4; TNF-α—Tumor Necrosis Factor-Alpha; IL-6—Interleukin-6; IL-10—Interleukin-10.

In conclusion, the effects of UFAs and SFAs on macrophage function and phenotype depend not only on the receptor involved, but also on other microenvironmental signals (Figure 3, Table 1; Table 2), as shown in in vitro and in vivo animal studies. Nevertheless, it is challenging to unambiguously assess the translational relevance of these data. Due to a diverse nature of inflammation and complex interactions between immune cells, mediators and signaling pathways, the influence of dietary FAs in in vivo interventions in humans may vary from the data obtained from in vitro and animal models. The effects of dietary FAs on specific cell populations are rarely reported as outcomes in clinical trials, and so it is difficult to link the clinical result with the cellular phenotype. Macrophages play a role in the pathogenesis of many diseases including asthma, allergic rhinitis, atopic dermatitis, food allergy, rheumatoid arthritis, multiple sclerosis, systemic lupus erythematosus, and type-1 and type-2 diabetes. We discuss the effects of dietary FAs in these diseases in Section 4.

### 2.3. Dendritic Cells

Dendritic cells (DCs) are central players in innate and adaptive immune responses [146]. They can recognize an antigen, process it, and further present and orchestrate T cell responses [146,147] as professional antigen-presenting cells. Several subsets of DCs are described. Conventional DCs (mDC1 and mDC2) and plasmacytoid DCs (pDCs) are present in the peripheral blood. Additionally, DCs are found in tissues such as skin, nose, lungs, stomach, and intestine. In in vitro models, DCs can be differentiated from blood monocytes or bone marrow and are referred to as monocyte-derived (mo-DCs) or bone marrow-derived DCs (BM-DCs), respectively [147,148]. All of these subpopulations vary in functions, secreted cytokines, and downstream effects. DCs shape immune responses by the release of essential cytokines such as IL-12, IL-6, TNF-α, chemokines, and interferons, for example, interferon-alpha (IFN-α) [146,147,148].

Mo-DCs stimulated with DHA, EPA, and ALA show a decrease in the ratio of activated CD1a^+^ cells and the expression of the GPR120 receptor [55]. Additionally, DHA and EPA downregulate the expression of major histocompatibility complex class II (HLA-DR) and costimulatory molecules (CD40, CD80, CD83, CD86) on the surface of DCs [56,57,58]. DHA-induced, TLR2, 3, 4, and 9-dependent reduction of pro-inflammatory cytokines, such as IL-12p70, IL-23, IL-27, is mediated by PPARγ activation and subunit 65 of NFκB transcription complex inhibition [56]. Interestingly, AA or EPA-treated mo-DCs have reduced the expression of TNF-α and IL-12p40 as compared to the cells stimulated with SFAs or MUFAs (PA or OA) [58]. Therefore, the immunomodulatory effects of dietary FAs on DCs have a significant influence on T-cell responses. PUFA-treated DCs have weaker T-cell stimulating properties than the cells treated with PA or OA [58,75]. A weaker activation signal from PUFA-treated (EPA and AA) DCs leads to decreased IL-2 and IFN-γ production by T cells [58]. N-3 and *n*-6 PUFAs inhibit DCs functions by blocking their responses to LPS, and this effect is independent of NF-kB activation and eicosanoid synthesis [58]. Treatment with DHA of BM-DCs, decreases their maturation, expression of costimulatory molecules, and production of cytokines (IL-12p70, IL-6, and IL-23) [75]. The study by Kong et al. showed that DHA-treated DCs fail to stimulate the proliferation of antigen-specific T cells and their Th1/Th17 differentiation in vitro and in vivo [75]. This phenomenon is accompanied by an increase in cyclin-dependent kinase inhibitor 1B cell cycle arresting agent, decrease in Tbet, GATA3, and RORγT transcription factors for Th1, Th2, and Th17 cells, which leads to the suppression of experimental autoimmune encephalomyelitis (EAE) [75]. Interestingly, T cells cocultured with DCs in the presence of DHA show a higher expression of tumor growth factor-beta and FoxP3, but not functional regulatory phenotype [75]. In addition, T cells activated by DHA and EPA stimulation increases in IL-13 and decreases in IFN-γ production in the mixed DC-lymphocyte reaction [34]. Loscher et al. demonstrated that conjugated linoleic acid (c9, t11-CLA) enhances the expression of IL-10 in DCs, at the same time suppressing the production of IL-12 [84]. Interestingly, the observed decrease in IL-12 is dependent on IL-10 [84]. C-9, t11-CLA also increases the expression of IL-10 receptor and activation of ERK and NF-κB that is necessary for the IL-10-mediated suppression of IL-12 production [84]. However, IL-10-dependent anti-inflammatory mechanisms of PUFA-stimulated DCs are yet to be clarified. Zapata-Gonzales et al. showed that mo-DCs differentiated in the presence of DHA increases the expression of CD36, HLA-DR, CD83, and CD86, decrease the expression of CD1a and reduce the secretion of both pro-inflammatory (IL-12p70, IL-6) and anti-inflammatory (IL-10) cytokines [76]. This effect is mediated by PPARγ: retinoic acid receptor (PPARγ:RXR) heterodimer [76]. Similarly, the treatment of mo-DCs with DHA and EPA leads to the inhibition of both TNF-α and IL-10 production and the ability of DCs to induce T cell proliferation [34]. Interestingly, DCs-dependent anti-inflammatory potency of PUFAs relies on the chemical structure of FAs (number of double bonds) [34].

SFAs tend to activate DCs. Lauric acid upregulates the expression of CD40, CD80, CD86, major histocompatibility complex class II, and pro-inflammatory cytokines release (IL-12p70, IL-6) in the BM-DCs [104]. Mo-DCs stimulated with PA show endocytosis of TLR4, cell maturation, and activation represented as the increased expression of CD86 and CD83 [98]. Additionally, the PA-induced TLR4-dependent secretion of IL-1β from DCs and the NFκB canonical pathway together with reactive oxygen species (ROS) generation participate in this mechanism [98]. In contrast to DHA, DCs treated with lauric acid show increased TLR4 activity and T cell activation capacity [104]. However, some studies failed to demonstrate the pro-inflammatory effects of SFAs on DCs [57,58].

The in vivo actions of dietary FAs on DCs have been investigated in the mouse model of IBD, where animals were fed either high (HFD)- or low-fat diet [133]. HFD decreases tolerogenic (CD11c^+^ CD103^+^ CD11b^+^) and increases pro-inflammatory (CD11c^+^ CD103^−^ CD11b^+^) DCs subpopulations in the gut [133] pointing it as an important factor in IBD pathogenesis [133]. SFA intake is also related to inflammasome-mediated inflammation [134]. In the mouse fed with HFD (45% PA), SFAs prime TLR4- dependent NLRP3-inflammasome activation and IL-1β secretion from BM-DCs [134]. On the contrary, rats fed with HFD-containing fish oil (*n*-3 PUFAs), but not safflower oil (*n*-6 PUFAs), show decreased antigen-presenting properties, a reduced proportion of CD2^+^ cells and the reduced expression of CD18, CD11a, HLA-DR, CD54 on the surface of lymph-borne DCs [111]. N-3 PUFA supplementation decreases the expression of CD80 and CD11c, the production of TNF-α, and phagocytosis in splenic DCs [112]. Likewise, in the mouse model of food allergy, van den Elsen et al. found the protective impact of fish oil supplementation [113]. Observed effect is mediated by reduced Th1-inducing (CD11c^+^ CD11b^−^ CD8α^+^) lymphoid DCs and increased tolerogenic, myeloid (CD11c^+^ CD11b^+^ CD8α^−^) DCs subpopulations [112,113]. In vivo anti-inflammatory actions of dietary *n*-3 PUFAs are also shown as the inhibition of T cell proliferation and the production of IFN-γ and IL-17 [114].

**Figure 4:**
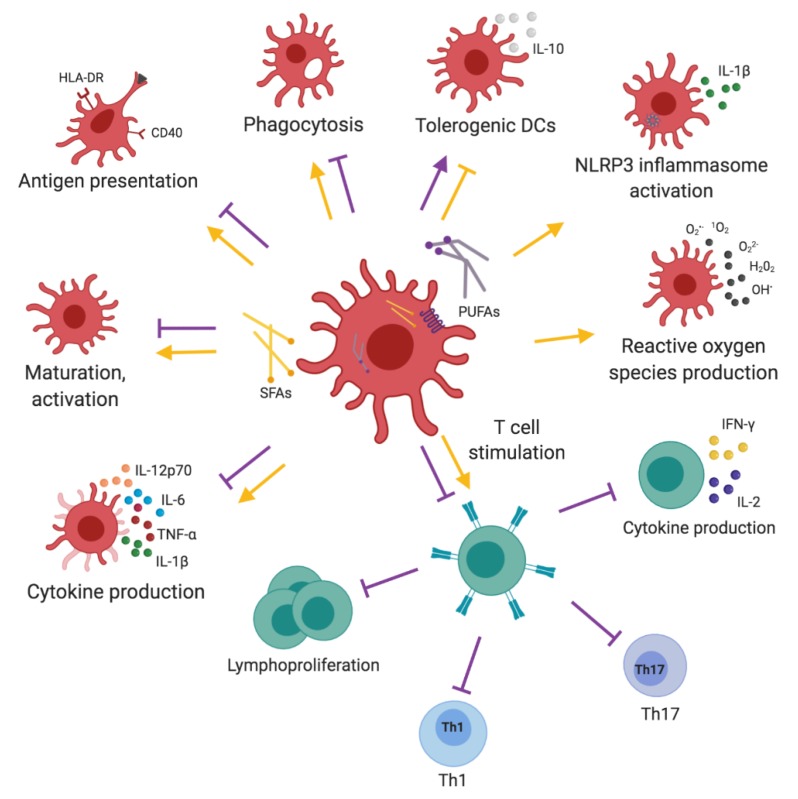
Anti-inflammatory and pro-inflammatory effects of dietary fatty acids on dendritic cells and dendritic cell-mediated T cell responses. For details, see the text. ⊥—inhibition; ↑—activation; PUFAs—Polyunsaturated Fatty Acids; SFAs—Saturated Fatty Acids; IL-10—Interleukin-10; IL-1β—Interleukin-1 beta; IFN-γ—Interferon-Gamma; IL-2—Interleukin-2; IL-12p70—Interleukin-12p70; IL-6—Interleukin-6: TNF-α—Tumor Necrosis Factor-Alpha; Th17—Type 17 T Helper Cells; Th1—Type 1 T Helper Cells; HLA-DR—Human Leukocyte Antigen DR Isotype; CD40—Cluster of Differentiation 40.

To summarize, PUFAs, especially *n*-3 PUFAs, inhibit the pro-inflammatory phenotype of dendritic cells and DC-mediated T cell responses. In contrast, SFAs increase DC maturation, activation, and T-cell stimulation properties (Figure 4, Table 1; Table 2). Results derived from in vitro studies and animal models generally allow for the in-depth understanding of the involved mechanisms. However, the clinical relevance of such results in humans needs to be interpreted with caution, due to a lack of relevant clinical trials.

### 2.4. Neutrophils

Neutrophils constitute the most abundant population of white blood cells physiologically distributed in two pools: (i) circulating cells representing 50–70% of the leukocytes in adult human blood, and (ii) marginated cells distributed in the organs such as bone marrow, spleen, liver, and lungs [149,150]. As an important part of innate immunity, they play essential roles in antimicrobial defense by direct (phagocytosis, degranulation, generation of neutrophil extracellular traps), or indirect (cytokine production) mechanisms [151]. In addition, neutrophils are involved in the activation of other innate and adaptive immune cells, resolution of inflammation, and tissue regeneration [150,151].

N-3 PUFAs are considered to exert anti-inflammatory roles in the course of several disorders, such as cardiovascular disease, rheumatoid arthritis or asthma, and observed beneficial effects are related with their immunomodulatory functions on neutrophils [152]. In vitro models are widely used for investigation of biological functions of FAs on neutrophil homeostasis. It has been shown that *n*-3 PUFAs compete with AA for lipoxygenase enzymes, as a result decreasing the production of highly inflammatory eicosanoids such as leukotriene B4 [152]. In addition, the oxidation process may be essential in the anti-inflammatory effects of *n*-3 PUFAs (EPA) on neutrophils [60]. It has been shown that the inhibition of neutrophil and monocyte adhesion depends on the oxidized-EPA-dependent activation of nuclear receptor PPAR-α [60]. Interestingly, the pro-inflammatory effects of PUFAs on neutrophils have also been reported. GPR84 is abundantly expressed on the surface of neutrophils and monocytes [153,154,155]. Sundqvist et al. showed that the activation of GPR84 triggers responses such as chemotaxis, ROS production, granule-localized receptor mobilization, and the activation of NADPH-oxidase in phagocytes [153]. Similarly, in the *n*-3 PUFA-stimulated neutrophils, an increase in ROS production, adhesion, and degranulation have been reported [152]. Both EPA and DHA increase the generation of ROS and TNF-α, but only DHA can enhance phagocytosis, antifungal properties, and IL-1β secretion by neutrophils [59]. Additionally, Khan et al. showed that dietary SFAs and MUFAs can induce NADPH-oxidase-dependent neutrophil extracellular traps formation in human neutrophils in vitro [94]. To explain these contradictory effects of various PUFAs on neutrophil biology, it has been hypothesized that the ratio of *n*-6/*n*-3 PUFAs, rather than *n*-3 PUFAs amounts, is essential to exert anti-inflammatory effects [152].

*N*-6 PUFAs are known from their pro-inflammatory properties in neutrophils. For AA, it has been shown as an increase in ROS production dependent on calcium signaling [86]. Additionally, n-6 PUFAs (AA) enhance the expression of tumor necrosis factor receptors: TNFR1 and TNFR2 on the surface of neutrophils [88]. The observed effect involves protein kinase C (PKC), ERK1/2, and cPLA_2_ [88]. Moreover, the reduced production of ATP, increased LDH release [89], mitochondria depolarization and accumulation of lipids [90] represent the cytotoxic effects of *n*-6-PUFA on neutrophils [89,90]. In in vivo settings, LA increases the marginated pool of neutrophils in the tissue by the induced expression of adhesion molecule L-selectin and the secretion of cytokines (KC/CXCL1, CINC-2αβ, CXCL3) [150,156,157]. On the other hand, LA complexed with the anti-inflammatory molecule α_1_-antitrypsin reduces LPS-induced IL-1β secretion in neutrophils. These anti-inflammatory properties of LA are likely to be mediated by reduced TLR2, TLR4, NLRP3 and caspase-1 expression and the activation of PPAR-γ [85]. Not only *n*-6 (LA) but also *n*-9 (OA) UFAs stimulate ROS production in neutrophils [86,91]. For OA, this effect is dependent on increased intracellular calcium concentration, leading to PKC activation [86]. Accordingly, OA increases phagocytosis, killing capacities, and cytokine production (VEGF, CXCL3, IL-1β) in neutrophils [91,92,93].

The distinct effects of dietary FAs have been widely investigated also in in vivo models. Strandberg et al. showed that diet rich in SFAs (HFD-S) decreases survival and increases bacterial proliferation in septic mice, due to an impairment of neutrophils/granulocytes functions [135]. In detail, HFD-S decreases the frequency of blood neutrophils, phagocytosing granulocytes, and ROS production [135]. Interestingly, mice fed with PUFA-enriched high-fat diet (HFD-P) for eight weeks have lower mortality and decreased bacterial load during *Staphylococcus aureus* sepsis [115]. The protective effect of PUFAs is also observed in uninfected mice, fed with HFD-P, shown as a higher frequency of neutrophils in the bone marrow and on the site of ongoing inflammation [115]. The anti-inflammatory effects of PUFAs in the course of bacterial infection depend on the diet rich in *n*-3 PUFAs [158,159]. On the other hand, *n*-3 PUFAs can also boost proinflammatory responses in neutrophils, as shown in mice genetically susceptible to colitis [121]. In this study, mice fed with fish oil show exacerbation of the clinical symptoms represented as the increased inflammation (enhanced numbers of systemic and local neutrophils), reduced thickness of mucus layer and goblet cell number in the cecum and colon [121]. Additionally, the proinflammatory effects of dietary fish oil were demonstrated as increased frequency of CD11b^high^, Ly6G^high^, and MHC class II^high^ neutrophils in the blood [116]. Those contradictory observations might partly result from the complexity of food supplementation and/or the kinetics of dietary FAs. Short treatment with LA leads to the increased expression of IL-1β and cytokine-induced neutrophil chemoattractant-2 alpha beta (CINC-2αβ), whereas, prolonged stimulation shows an opposite effect of the reduced secretion of those cytokines [156].

The relevance of *n*-3 PUFA supplementation in anti-inflammatory functions of neutrophils has been confirmed in an intervention study in humans, where patients with chronic kidney disease were supplemented for eight weeks with *n*-3 PUFAs (mainly EPA and DHA) [117]. Increased neutrophil release of several specialized pro-resolving mediators such as EPA-derived 18-hydroxyeicosapentaenoic acid, resolvins E1, E2, and E3 and DHA-derived 17-hydroxydocosahexaenoic acid and resolvin D5 is accompanied with decreased plasma myeloperoxidase levels [117]. Moreover, the same study group report that supplementation with *n*-3 PUFAs is associated with a significant increase in neutrophil telomere length, possibly due to decreased oxidative stress [118]. Additionally, the effect of DHA-rich fish oil supplementation has been studied during acute exercise in wheelchair athletes [119]. Intake of *n*-3 PUFAs restores their initially impaired neutrophil functions [119]. Similarly, parenteral infusion with *n*-3, but not *n*-6 PUFAs, leads to partial restoration of neutrophil functions impaired by sepsis [160]. Additionally, patients undergoing cancer chemotherapy significantly benefit from low dose fish oil supplementation, which is clinically demonstrated as an increase in body weight. Mechanistically this effect is related to an increase in neutrophil numbers and improvement of their functions [120]. Importantly, *n*-3 PUFAs can also influence immune development in early life [25,26,161]. In contrast, several other interventional studies failed to prove the positive effects of *n*-3 PUFA supplementation on neutrophil-dependent immune functions [162,163,164], suggesting that the inclusion criteria, measured outcomes, as well as the dose and form of supplementation, may vary between the studies and should be unified in the future. Additionally, the age and gender of individuals recruited to the study are also significant factors to be concerned in the experimental set up [121,165,166].

**Figure 5:**
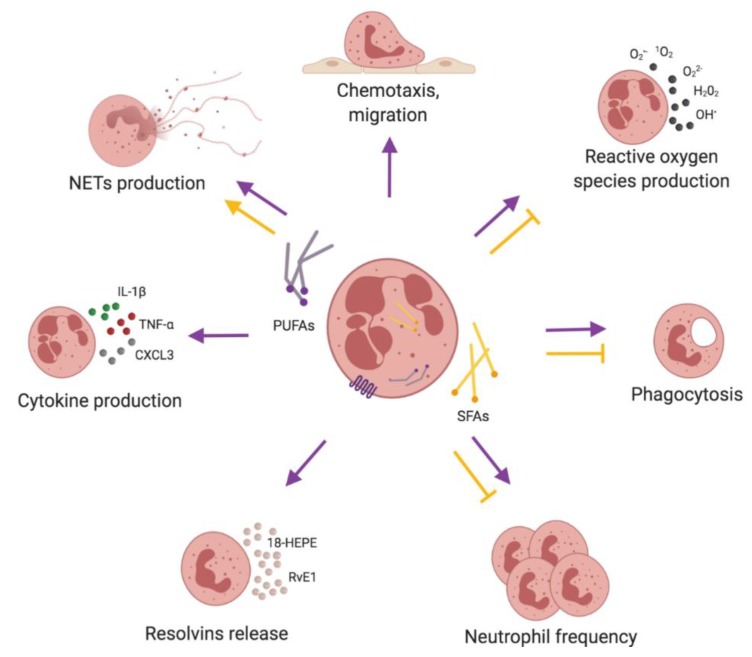
Pro-inflammatory and anti-inflammatory effects of dietary fatty acids on neutrophils. For details, see the text. ⊥—inhibition; ↑—activation; PUFAs—Polyunsaturated Fatty Acids; SFAs—Saturated Fatty Acids; 18-HEPE—18-Hydroxyeisostatetraenoic Acid; Rve1—Resolvin E1; IL-1β—Interleukin-1 Beta; TNF-α—Tumor Necrosis Factor-Alpha; CXCL3—Chemokine (C-X-C Motif) Ligand 3; NETs—Neutrophil Extracellular Traps.

In summary, evidence coming from in vitro and animal models allows us to conclude that PUFAs increase and SFAs decrease pro-resolving functions of neutrophils, restoring balanced innate immune responses (Figure 5, Table 1; Table 2). However, the data from clinical trials are inconsistent (summarized in the section) [117,118,119,120,160,162,163,164] and require further confirmation.

### 2.5. Innate Lymphoid Cells

Innate lymphoid cells (ILCs) have been divided into three subpopulations—ILC1, ILC2, and ILC3—based on the expression of transcription factors, membrane molecules, and cytokine profiles [167,168]. ILC3s are further subdivided into two groups: (i) natural-cytotoxicity-receptor-positive ILC3 (NCR^+^ ILC3) and (ii) phenotypically mimicking fetal lymphoid tissue-inducer ILC3 cells (LTi-like ILC3) [169]. Due to the broad spectra of secreted cytokines, ILCs have diverse, significant immunomodulatory properties and have a role in both the protection and progression of various diseases [167,168].

ILCs significantly contribute to the homeostasis of adipose tissues [168]. The effects of dietary nutrients, such as tryptophan metabolites, vitamin A and retinoic acid, on ILCs population, have been widely investigated [168,170,171,172,173]. Due to the expression of lipid receptors, ILCs possess the potential to respond to dietary FAs [174,175]. However, this issue has not been studied in sufficient detail. ILC3 express GPR183 receptor, which can recognize cholesterol metabolites (7α,25-hydroxycholesterol; 7α,25-OHC) [175]. Emgard et al. showed that 7α,25-OHC, synthesized in the intestine, increases recruitment of LTi-like ILC3 into intestinal lymphoid structures, contributing to the pathogenesis of IBD [175]. On the other hand, FAs metabolism is essential for the protective functions of ILC2 during helminth infection [174]. The study by Wilhelm et al. showed that all subpopulations of ILCs can acquire long-chain FAs from the environment, with the highest potency in the ILC2, followed by ILC3 and ILC1 [174]. FAs protect the intestinal barrier during helminthiasis by significant expansion of ILC2 population [174]. Interestingly, throughout vitamin A deficiency, uptake of FAs by ILC2 increases, filling up the metabolic gap and assuring the maintenance of protective response [174]. Based on these limited reports, we present here the unmet need for further research on the influence of dietary FAs on ILCs properties. This might lead to a better understanding of immune mechanisms and the development of novel therapeutic approaches.

## 3. Dietary Fatty Acids and Adaptive Immune Responses

### 3.1. T Cells

T lymphocytes are the main players in cellular adaptive immunity. They originate in the bone marrow and complete their maturation in the thymus, where they begin to express T cell receptors (TCRs). T cells are considered immunologically naïve until they encounter MHC–peptide complexes, for which their TCRs have high affinity. This activation leads to T cell proliferation and differentiation into effector cells. After the initial expansion, most of the effector T cells die by apoptosis, but a part of them survives, becoming memory T cells. The main types of mature T cells consist of helper T cells, expressing CD4 coreceptor, and cytotoxic T cells expressing CD8 coreceptor. Within these two main types, many subspecialized subsets play a crucial role in immune memory and tolerance, including T helper (Th1, Th2, Th17, Th9, Th22, Tfh) and T regulatory (Treg) cells [176,177].

It has been largely described that dietary FAs influence T cells by modifying their fate and/or modulating their effector functions. FAs can passively diffuse through the T cell membrane and bind to a cytoplasmic protein named Fatty Acid Binding Proteins (FABPs). FABPs promote nuclear localization of FAs and activate nuclear receptors PPARs, enhancing their activation [178,179]. In particular, T cells express FABP4 and 5, described to be involved in uptake, transport, storage, and metabolism of FAs [180]. In addition to this mechanism, FAs can bind to GPRs expressed on the plasma membrane. Both T and B lymphocytes express GPR84, enabling the binding of medium-chain FAs, such as lauric acid [155,181].

Several in vitro studies have demonstrated that UFAs can exert immunosuppressive effects on T cells. When added before stimulation, UFAs can reduce the proliferation of T cells in a dose-dependent manner, by acting on early T cell signal transduction. The reduced T-cell proliferative capacity, observed in the presence of dietary DHA and EPA, is always accompanied by a reduction in IL-2 secretion [61,62,63,64] or by the reduction of its receptor IL-2R [63,64,67,182], suggesting that dietary *n*-3 PUFAs can interrupt the autocrine IL-2 pathway. Moreover, DHA and EPA can regulate Janus Kinases—Signal Transducer and Activator of Transcription Proteins (JAK-STAT) pathway by decreasing JAK1 and JAK3 phosphorylation with the subsequent inhibition of STAT5 phosphorylation [67], and by inhibiting Protein Kinase B (Akt) and ERK1/2 phosphorylation [67,68]. In addition to IL-2 signaling, EPA and DHA can modify the expression of other cytokines in T cells. Several studies have shown a dose-dependent reduction of TNF-α, IL-4, and IL-10 in the presence of UFAs [64,65,66]. Furthermore, DHA and AA decrease T-cell proliferation in a DC and T cell co-culture system by increasing the proportion of T cells expressing FoxP3, thus promoting the induction of regulatory T cells [77]. Moreover, studies in human T cell lines have shown that DHA exerts immunosuppressive properties by modulating calcium concentration, recruiting calcium from the intracellular pool, and opening calcium release-activated calcium channels [78,79,80]. PUFAs-induced calcium increase has also been observed in rat T cells [81,82], but how it affects T cell activation remains to be elucidated. Interestingly, UFAs can cause cell death in T lymphocytes. High doses of the monounsaturated OA induce apoptosis through the activation of the caspase-3 pathway, while the PUFAs LA and EPA can induce apoptosis by involving ROS production [63,87,183].

While UFAs have shown anti-inflammatory effects, SFAs, such as PA, have been described as an essential factor driving T-cell activation. PA-treated T cells show the activation of Phosphoinositide 3-kinase/Akt signaling, a pathway known to be rapidly activated upon T-cell priming [99,100,101]. In addition, PA treatment significantly upregulates, via JAK/STAT5 pathway, the expression of signaling lymphocyte-activation molecule family member 3, a receptor strongly associated with T cell activation and increases transcription of several inflammatory genes, including TNF-α, IL-1β, IL-6, IFN-γ, IL17A and IL-2 [100,102]. T cells cultured in the presence of PA show the increased expression of the inflammatory marker CD69 [102] and reduced levels of C-C chemokine receptor type 7 and L-selectin [99], suggesting the acquisition of an effector phenotype. Like PA, lauric acid has been described to have pro-inflammatory properties in vitro. In fact, in T cell cultures, it increases IL-2 expression and presents an additive effect on IL-17 and granulocyte-macrophage colony-stimulating factor, thus enhancing the differentiation of Th17 [105]. In contrast, it reduces Th2 differentiation [105].

In vivo, as in vitro, UFAs show rather immunomodulatory and immunosuppressive properties. In mice, a diet enriched with EPA and DHA reduces the proliferation of T cells, by downregulating IL-2 secretion and interleukin-2 receptor alpha chain expression, and decreases the intracellular production of phospholipid-derived second messengers, essential for cell activation, such as diacylglycerol and ceramide, as well as the level of phospholipase Cγ [122]. These observations suggest that the diet rich in UFAs may affect the intracellular signaling cascade, upstream of these second messengers, and thus may involve membrane-proximal events. This hypothesis is consistent with another study with an animal model, where PUFA-enriched diet modified the plasma membrane topography in CD4^+^ T cell and disrupted the spatial organization of another critical second messenger, phosphatidylinositol 4,5-biphosphate, thereby perturbing downstream signals required for T cell proliferation [123]. Furthermore, a diet rich in *n*-3 PUFAs can reduce pro-inflammatory T cells in most of the fat tissues, since it modulates the migration properties of T cells. This is achieved by regulating the expression of chemokine receptors on T cells, such as C-C chemokine receptor type 4 and C-X-C chemokine receptor type 4 [66], and also by a transcriptional decrease in the expression of adhesion molecules such as P-selectin and intracellular adhesion molecule 1 on the endothelium [124]. In addition, PUFAs can affect the motility of CD4^+^ T cells by interfering with the cytoskeletal rearrangements required for cell migration. It has been described that EPA reduces in vivo the formation of pseudopods in T cells and decreases the ratio between F-actin and G-actin, together with the downregulation of Rhoα and Rac1, two small Rho GTPases involved in cell migration [125]. In a mouse model of arthritis, *n*-3 PUFAs decrease IL-6, IL-23, and IL-17 expression, while they stimulate the expression of FoxP3 and thus the differentiation of regulatory T cells [126,127]. The same induction of regulatory T cells by *n*-3 PUFAs has been observed in mouse models of atopic dermatitis [184,185]. Nevertheless, the effect of UFAs on regulatory T cells seems to be controversial. DHA dose-dependently reduces the capacity of regulatory T cells to inhibit the effector T-cell proliferation by downregulating ERK1/2 and Akt phosphorylation and upregulating histone deacetylase and cyclin-dependent kinase inhibitor 1B expression. On the other hand, it can also upregulate the mRNA expression of FoxP3, cytotoxic T-lymphocyte-associated protein 4, tumor growth factor-beta, and IL-10, known to be important for the immunosuppressive activity of regulatory T cells [66].

On the contrary, in mice fed with an SFA (PA)-enriched diet, CD4^+^ memory T cells show upregulation of C-X-C motif chemokine receptor 3 (CXCR3), and downregulation of C-C chemokine receptor type 7 and L-selectin, thus trafficking into non-lymphoid tissues and inflammatory sites [99]. CXCR3^+^ memory T cells accumulation in fat sites has also been observed in obese humans [99]. In an EAE mouse model, a diet rich in either lauric or palmitic acid leads to a more severe course of the disease, with enhanced Th1 and Th17 cell differentiation and increased T cell infiltration into the central nervous system [101,136]. In a study with healthy human subjects, circulating SFAs, such as PA and MA, were positively associated with several markers of inflammation, such as C-reactive protein, IL-1 receptor antagonist, IFN-γ, and C-C chemokine ligand 5 [137].

**Figure 6:**
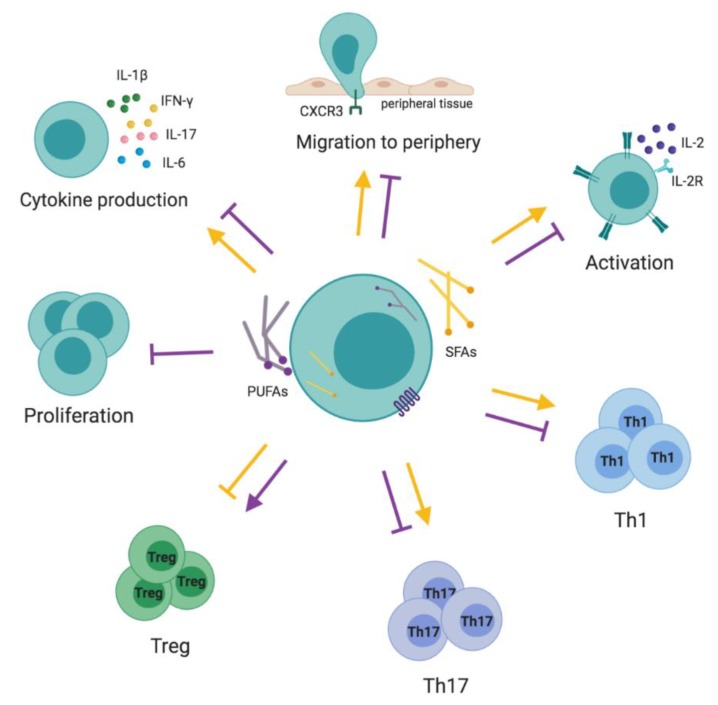
Anti-inflammatory and pro-inflammatory effects of dietary fatty acids on T cells. For details, see the text. ⊥—inhibition; ↑—activation; PUFAs—Polyunsaturated Fatty Acids; SFAs—Saturated Fatty Acids; Th1—Type 1 T Helper Cells; Th17—Type 17 T Helper Cells; Treg—T Regulatory Cells; IL-1β—Interleukin-1 Beta; IFN-γ—Interferon-Gamma; IL-17—Interleukin-17; IL-6—Interleukin-6; IL-2—Interleukin-2; IL-2R—Interleukin-2 Receptor; CXCR3—Chemokine Receptor CXCR3.

In summary, PUFAs increase Treg numbers and decrease T cell proliferation, activation and differentiation into Th1/Th17 phenotypes. In contrast, SFAs lead to T cell activation and differentiation into proinflammatory T cell phenotypes (Figure 6, Table 1; Table 2). Notably, the presented conclusions are based mainly on in vitro and animal studies. Much less is known about the clinical relevance of T-cell-mediated responses after PUFA supplementation. Several inconsistent reports are available in the field, showing positive [186,187,188,189] or null [190,191,192,193] results. Therefore, definite conclusions about the potential protective or harmful effects of PUFA stimulation in T cells cannot be stated.

### 3.2. B Cells

B lymphocytes mediate the humoral adaptive immune response. They originate and maturate in the bone marrow and, with the assistance of helper T cells, can differentiate into plasma cells, able to produce and secrete antibodies. Antibodies bind to antigens, inducing their neutralization, lysis, or phagocytosis. B cells can also secrete several cytokines, chemokines, and other mediators and take an active part in the immune response beyond antibodies production. To date, the impact of FAs on B cell function is rather poorly studied. Data obtained thus far are mostly from in vitro studies of B cell lines, and observations made in several mice models seem to be quite contradictory.

In vitro, SFAs (PA) can suppress B-cell activation through lipoapoptosis, but the presence of OA and DHA prevents this effect [103]. Exposure of Raji B cells to EPA and DHA decreases the production of the key immunoregulatory cytokines, such as IL-10, TNF-α, and INF-γ [69]. In mouse primary B cells, DHA exposure decreases cytokine production, such as IL-6, after LPS stimulation [103]. In Ba/F3 cells, a pro-B cell line, lauric acid induces dimerization, and recruitment of TLR4 to lipid rafts. Lipid rafts are ordered and tight microdomains of the plasma membrane that serve as a signaling platform to concentrate receptors, coreceptors, adaptors, and downstream signaling molecules to promote signal transduction. The presence of *n*-3 PUFAs (DHA) can inhibit TLR4, probably by altering the membrane FAs composition and the formation of the lipid rafts, thus reducing the recruitment of signaling molecules [83].

In vivo, *n*-3 PUFAs have shown immune-enhancing properties on B-cell activities in several mouse models. In mice fed with a EPA- and DHA-enriched diet, B cell-mediated responses seem to be boosted, with the increased expression of the activation markers CD69 and CD40 [103,128], upregulation of B-cell cytokines IL-6, IFN-γ, TNF-α and IL-10 [103,128,129] and an increase in the percentage of splenic transitional and marginal zone B cells, and peritoneal B1 cells. Furthermore, the upregulation of immunoglobulin M in the spleen as well as in serum has been observed [130,131]. In addition, analyses of B cells from SMAD-deficient mice have shown an increase in the expression of plasma level of type-2 cytokines, playing a role in B function regulation including IL-5, IL-13, and IL-9, and an increase in caecal immunoglobulin A, when compared with control mice [128]. Interestingly, DHA, but not EPA, increases monosialotetrahexosylganglioside surface expression and changes the lipid composition of B cells, increasing the size, the order, and the distribution of rafts, subsequently affecting protein clustering and downstream signaling [128,129,132] (Table 1; Table 2). In summary, limited research in the field does not allow to draw any reliable conclusions regarding the immunomodulatory effects of dietary FAs on B cells homeostasis.

## 4. Dietary Fatty Acids in Immune-Related and Metabolic Diseases

### 4.1. Allergic Diseases

The prevalence of allergic diseases has increased rapidly in recent years. It is currently accepted that various environmental exposures, lifestyle factors, and diet play a role in this increase in allergic disease morbidity [194]. In recent years, a causal link between dietary FAs and allergic diseases has often been investigated [25]. Most of the studies focused on the preventive or therapeutic effects of PUFAs. *N*-3 and *n*-6 PUFAs are essential components of the phospholipids in cell membranes and, among others, significantly participate in the modulation of allergic responses via their direct effects and also by their key metabolites, including eicosanoids [195]. Considering that LA and ALA compete for the receptors, a diet rich in *n*-6 PUFAs increases the *n*-6/*n*-3 PUFA ratio. It causes an increase in AA-derived metabolites, which in turn may enhance or resolve allergic inflammation, depending on the downstream pathways and receptors [196]. Conversely, with an increase in *n*-3 PUFAs-intake in the diet, EPA and DHA might compete with AA for COX, lipoxygenase, and cytochrome P450 pathways. As a result, more specialized pro-resolving mediators, such as lipoxins, protectins, marensins are produced [195]. Additionally, a higher *n*-6/*n*-3 PUFA ratio influences the balance of Th1/Th2 cells via IL-13-inhibition [196]. Thereby, an imbalance in the consumption of PUFAs is linked with the protection or risk of the development of allergic diseases. We will discuss below the complex interactions between dietary FAs and asthma, allergic rhinitis, atopic dermatitis and food allergy.

#### 4.1.1. Asthma

Asthma is a common, heterogeneous, chronic inflammatory disease of the airways with the symptoms of recurrent reversible bronchial obstruction. The disease has diverse clinical phenotypes and molecular pathogenesis, and it affects more than 300 million people worldwide [197]. Many epidemiological studies show that the prevalence of asthma is related to the dietary habits, often assessed as an intake ratio of SFAs/PUFAs. Higher risk of asthma is reported in populations with an increased consumption of SFAs- and *n*-6 PUFA-rich food [47,198,199,200,201,202]. Accordingly, lower intake of *n*-3 PUFAs leads to worsening of asthma and lower pulmonary function [203]. In contrast, in populations with higher intake of *n*-3 PUFAs, such as Inuits, asthma prevalence is significantly reduced [204,205,206]. Inuits are indigenous population of arctic and sub-arctic regions of Greenland, Canada and Alaska, whose diet is based mainly on fish and arctic marine mammals and hence is enriched with *n*-3 PUFAs [207]. Interestingly, there is an association between genotype and disease development in geographically separate Inuit populations—one living in Greenland and the other, migrated to Denmark [208]. This study concluded that local environment (higher pollution and aeroallergens rate in Denmark) and lifestyle changes (lower *n*-3 PUFA intake) increase incidence of asthma in the urban population with similar genetic background [208]. However, in light of the recent results, demonstrating the importance of obesity and microbiome in such studies [194,209,210,211,212,213], the conclusions should be carefully interpreted.

Furthermore, many studies noted that maternal intake of fish or *n*-3 PUFAs during pregnancy has beneficial effects on allergic outcomes in the offspring and prophylactic potential for preventing asthma [136,196,214]. However, duration of the mother-derived protection is controversial. Breast milk containing higher amounts of *n*-3 PUFAs has been found to be protective against allergic diseases, both in early childhood and up to the age of 14 years [196]. Yet, another follow-up study reported that these protective effects become weak at the age of 6 years [215]. In a Danish cohort, as well as in the animal models [216], both *n*-3 and *n*-6 PUFA intake during pregnancy show protective effect against asthma and allergic sensitization in young children [217]. Interestingly, *n*-6 PUFAs are also linked with a reduced prevalence of asthma and increased probability of remission between ages 8-16 years [218]. Due to the inconsistency in results and potential role in increased asthma prevalence, the hypothesis linking maternal *n*-3 PUFA intake to childhood allergic disease cannot unequivocally be confirmed or rejected [219]. Nevertheless, *n*-3 PUFA supplementation during pregnancy and breast-feeding may be protective for the children, of which mothers have low pre-existing levels of DHA and EPA [25].

Several more recent interventional clinical trials investigated the effects of PUFA supplementation on development and symptoms of asthma. Higher proportions of *n*-3 PUFAs in the diet are linked with a decreased risk and improved asthma control in both children [201,220,221,222,223] and adults [47,202,224,225,226,227,228,229,230,231,232,233,234]. In adulthood, benefits are demonstrated as a decreased prevalence of asthma symptoms, improved asthma control, decreased levels of fractional exhaled nitric oxide (FeNO), blood eosinophilia and improved lung function [47,202,224,225,226,227,228,229,230,231,232,233,234]. Additionally, *n*-3 PUFAs have beneficial effects by reducing airway hyperresponsiveness and severity of exercise-induced bronchoconstriction [233]. Interestingly, not only PUFAs, but also *n*-9 MUFAs (OA) and the high consumption of olive oil decrease the risk of asthma [235]. However, several other groups found that PUFA supplementation is not beneficial for pulmonary health [236,237,238,239,240,241,242,243,244,245,246,247]. Finally, *n*-3 PUFA intake in children can be also associated with an increased risk of wheeze [248], allergic rhinitis, eczema, and allergic sensitization up to 18 years of age and with reduced lung function at the age of 12 years [249] (Table 3).

The partial explanation of observed discrepancies in observational and interventional studies may result from the variations between the design of the studies and uncontrolled confounders. Asthma is a heterogenous disease, therefore several factors in the clinical characteristics of the study population should be taken into consideration. Phenotype, endotype, severity of the disease, existing comorbidities, daily diet, microbiome, occupational exposure, smoking status and treatment strategies (corticosteroid use) are crucial factors determining homogeneity of the study groups [194,212,213,250]. Additionally, differences in experimental design, inclusion criteria and methodology may lead to the differences in the reported findings. All future studies should consider unified inclusion and exclusion criteria and methodology, and account for additional essential variables including other nutrients and microbiome composition [25,26].

#### 4.1.2. Allergic Rhinitis

Allergic rhinitis (AR), chronic inflammation of the mucous membranes of the nose, is a common allergic disease with different phenotypes [251]. Limited number of studies has investigated the effects of dietary FAs on AR development. No evidence is supporting a protective association of fish consumption during pregnancy with AR from infancy to 8 years of age [252,253]. However, in children, early introduction of fish (before 9 months) reduces the prevalence of AR [254,255,256]. In addition, in a Swedish cohort, the regular consumption of oily fish and *n*-3 PUFAs in childhood decrease the risk of rhinitis, especially non-allergic rhinitis, between the ages of 8–16 and an increased *n*-6/*n*-3 PUFA ratio enhances this risk [251]. In adults, conflicting findings show both protective [257,258] and no effect [243] of PUFA supplementation (Table 3).

#### 4.1.3. Atopic Dermatitis

Atopic Dermatitis (AD) is a chronic inflammatory skin disease characterized by significant disruption of the skin barrier. *N*-3 and *n*-6 PUFAs provide an appropriate structure, elasticity, and functionality of cell membranes and are vital for the synthesis of intracellular lipids in the corneous layer in the epithelium. Supplementation with *n*-3 PUFAs during pregnancy leads to a decrease in incidence and intensity of AD in children [259,260]. In addition, infants with higher plasma levels of *n*-3 PUFAs have significantly reduced the prevalence of eczema [261]. In contrast, a diet low in *n*-3 PUFA intake is associated with an increased risk of AD development [262,263,264,265]. However, the studies are not consistent. In an Australian cohort, the prevalence of AD in infants consuming *n*-3 PUFAs for six months is similar to the control group [266]. Another study found that *n*-3 PUFA intake in pregnancy has no impact on the incidence and severity of AD [267].

The effect of dietary FAs on AD in adults has not been studied in sufficient detail. Solvoll et al. demonstrated no association between dietary habits and clinical status of adult AD patients [268] (Table 3). Clinical trials on the therapeutic effects of FAs on severity of AD are not conclusive. Some of the reports show beneficial effects [269,270,271,272,273], whereas in others supplementation with PUFAs does not improve disease outcome [274,275]. However, because of no adverse effects, supplementation with EPA and DHA, combined with regular treatment, is recommended for prevention and treatment of AD [25].

#### 4.1.4. Food Allergy

Food allergy, frequently developed early in life, has an increasing prevalence worldwide [276]. Supplementation with fish oil during pregnancy and lactation has been shown to reduce allergic sensitization to food proteins in the offspring [196,259,260,266,277,278,279,280,281]. However, mother-derived protection is only temporary [215]. The beneficial effect of *n*-3 PUFA supplementation can be maintained by early introduction of fish to the diet. Increased *n*-3 PUFA intake reduces sensitization to foods and development of allergic disease in infants [282]. Interestingly, this effect is observed only in children without any parental allergic history [282]. As in the case of other allergic diseases, the results of clinical trials are contradictory. Several studies on food allergy show no protection after increased PUFA supplementation [214,280,283,284,285,286,287,288]. However, in a Cochrane review, it is summarized that supplementation with *n*-3 PUFAs during pregnancy reduces sensitization to eggs in 12–36-month-old infants but does not make a significant difference in cow’s milk, wheat and peanut sensitivity [289] (Table 3).

In summary, clinical trials investigating the effects of dietary FAs in the course of allergic diseases show inconsistent results. Despite several discrepancies regarding protective effects, *n*-3 PUFA supplementation is not harmful. Therefore, essential guidelines on FAs consumption and diet diversity have been recently issued by international scientific societies [25,26]. Accordingly, *n*-3 PUFAs augmentation may be considered as beneficial supplementary therapy in course of allergic diseases and it is recommended to adhere to the respective countries’ guidelines [25].

### 4.2. Autoimmune Diseases

#### 4.2.1. Rheumatic Diseases

Rheumatoid Arthritis (RA) is a long-term autoimmune disorder characterized by chronic inflammation, destruction of the cartilage, pain, and swelling of the joints due to the pathological activation of various pro-inflammatory cells. Independent of the inflammation, higher *n*-3 PUFA intake is reported to be inversely associated with both unacceptable and refractory pain [321]. Additionally, *n*-3 PUFAs composition of synovial fluid correlates positively with *n*-3 PUFA intake and negatively with the pain score [291]. The majority of clinical trials investigating the effects of *n*-3 PUFA supplementation showed clinical benefits in patients with ongoing disease [293,295,321,322,323,324,325,326]. High *n*-3 PUFA intake protects against the incidence of RA [292,293,294] and improves RA prognosis [295,296]. Moreover, *n*-3 PUFAs decrease the duration of morning stiffness, reduce the number of tender and swollen joints in patients with RA [297]. End-point measurements described in the studies include broad spectrum of outcomes and varies between certain clinical trials. Number of tender and/or swollen joints, duration of morning stiffness, pain, grip strength, inverse association with anti-cyclic citrullinated peptide positivity and reduction in non-steroidal anti-inflammatory drugs (NSAIDs) intake are most commonly assessed benefits. The majority of clinical trials show improvement of some, but not all end-points and those results vary among studies. Negative results are also reported [327,328,329,330]. Weaknesses of the studies include different doses of EPA and DHA, varying study duration and small sample size. Additionally, some of the studies required changes in the on-going treatment before enrollment which may influence the study outcomes (e.g., reduced NSAIDs use) [322]. Despite several inaccuracies, a fairly high number of studies show that an *n*-3 PUFA-rich diet is beneficial in RA patients and may be considered as an addition to the regular treatment (Table 3). Daily supplementation with 3 g of EPA and DHA is recommended [331].

Patients with rheumatic diseases usually have lower levels of PUFAs in serum [290]. It has been demonstrated that serum lipid profile is also altered in other rheumatic diseases, such as polymyositis (PM), dermatomyositis (DM), and Sjögren Syndrome (SS) [298]. Based on the evidence from RA the effects of dietary FAs have been also investigated in patients with PM, DM and SS. Polymyositis and dermatomyositis are chronic autoimmune disorders characterized by skeletal muscle weakness, fatigue, and damage. While PUFAs have an important role in regulating skeletal muscle growth and functions, PA is associated with inflammation and atrophy of myotubules [298], and these adverse effects of PA may be alleviated by PUFA treatment [332]. Sjögren Syndrome is characterized by progressive lymphocytic infiltration of the salivary and lacrimal glands [299]. PA augments inflammation and induces apoptosis in epithelial cells of the salivary glands [333]. Although the clinical significance of FAs in the pathogenesis of Sjögren Syndrome is still unclear, some papers have reported that lipid-related molecules, such as the metabolites of *n*-3 PUFAs, block inflammation and increases barrier function in the cells of salivary glands [299,300] (Table 3).

Systemic lupus erythematosus (SLE) is a prototype of autoimmune diseases with no clear etiology. *N*-3 PUFAs have a positive impact on clinical conditions in SLE by reducing inflammatory mediators, the chemotaxis of leukocytes, and the expression of adhesion molecules [301]. A 16-week randomized controlled trial has established that *n*-3 PUFA supplementation improves endothelial function also in patients with an antiphospholipid syndrome characterized by endothelial dysfunction and recurrent thrombotic episodes [302] (Table 3).

#### 4.2.2. Multiple Sclerosis

Multiple Sclerosis (MS) is an inflammatory disease of the central nervous system, leading to demyelination and formation of focal lesions with lymphocyte infiltration [334]. An autoimmune basis of MS depends on the imbalance between pro-inflammatory and anti-inflammatory mechanisms [335]. Animal model of experimental EAE has noted the beneficial effects of FAs mediated by shifting macrophage polarization from an M1-like inflammatory phenotype to an anti-inflammatory M2-like phenotype, which in turn prevents de-myelinization, promotes neuroprotection and re-myelinization [306]. Similarly, to other autoimmune diseases, human studies regarding PUFA supplementation in MS are inconsistent. Some cohorts report the protective effects of high PUFA intake on MS development [303,304,305], whereas other demonstrate no significant clinical outcomes [336,337,338,339,340] (Table 3). Current evidence of beneficial response to *n*-3 and *n*-6 PUFA supplementation in patients with MS is controversial. More clinical trials are needed to reliably evaluate its potential use as a supportive therapy.

#### 4.2.3. Type 1 Diabetes

Type 1 Diabetes mellitus (T1DM) is an autoimmune disease represented by the destruction of pancreatic β-cells and characterized by early-onset, often in the infancy or childhood. Immune dysfunction precedes the disease onset, and *n*-3 PUFAs may play a role in the development of healthy immune responses in the early months of life [341]. A case-control study by Niinisto et al. showed that *n*-3 PUFAs are associated with a decreased risk of primary autoimmunity and can provide protection from T1DM in infants [307]. A study performed in older children reported that high *n*-3 PUFA intake is associated with a decreased risk of islet autoimmunity but is not associated with progression to T1DM [308]. Only one prospective randomized clinical trial was conducted in adult patients with T1DM [342]. Thirty-eight patients with T1DM with increased urinary albumin excretion were randomly assigned to decrease the ratio of SFAs/PUFAs to 1.0 by replacing SFAs with LA-rich products for two years. A diet supplemented with LA reduces atherogenic lipoprotein proteins but has no beneficial effect on renal dysfunction in patients with T1DM, and might even promote some abnormalities [342] (Table 3). More relevant clinical trials should be performed in the future for better understanding the mechanisms and clinical relevance of PUFA supplementation in T1DM patients.

### 4.3. Type 2 Diabetes

The prevalence of type 2 diabetes (T2DM), a metabolic disorder that accounts for 90–95% of all diagnosed cases of diabetes in adults, is sharply increasing worldwide, especially in low and middle-income countries, with obesity and aging seen as the main driving forces [343]. T2DM is characterized by hyperglycemia due to impaired insulin secretion from pancreatic β-cells and insulin resistance in insulin-sensitive tissues such as skeletal muscle, liver, and adipose tissue. The exact mechanism underlying insulin resistance remains elusive. Compelling evidence indicates that chronic low-grade inflammation, reflected by abnormal cytokine production, as well as the stimulation of intracellular inflammatory signaling pathways in insulin-sensitive tissues, is an essential component of this process [344]. In line with this, human data have revealed elevated plasma levels of both pro-inflammatory cytokines (such as TNF-α, IL-6, and IL-1β) and acute phase reactants (such as C-reactive protein, serum amyloid A, α1-acid glycoprotein, and fibrinogen) in T2DM patients [345,346]. Furthermore, the increased activation of NF-κB—a well-known transcription factor presents in all cell types, regulating the expression of pro-inflammatory genes encoding TNF-α, IL-6, and IL-1β, among others—has been found in diabetic skeletal muscle, which is thought to be a significant site for most insulin-stimulated glucose utilization in the body. Importantly, this abnormality is associated with the development of the insulin resistance state, confirming the existence of the link between inflammation and metabolic deregulation in T2DM [347]. The detailed mechanisms responsible for the enhancement of NF-κB signaling in diabetic skeletal muscle are not clear.

Mechanistic studies have revealed that increased intramyocellular accumulation of lipid metabolites, especially diacylglycerol and/or ceramide, derived from the increased plasma SFAs, such as PA, is responsible for this effect through the activation of the PKC-theta (PKCθ). PKCθ is the most abundant PKC isoform in skeletal muscles that functions as an upstream kinase of NF-κB activation. The consequence of this is the increase in the muscular NF-κB-dependent expression of pro-inflammatory cytokines, such as IL-6 and TNF-α, which negatively regulates insulin signaling [309,310].

Another mechanism attributed to the ability of SFAs to induce inflammatory-mediated insulin resistance in skeletal muscles is the stimulation of COX-2 expression by p38 MAPK that ultimately leads to the production of biologically active PGE_2_. It appears to be involved in inflammatory responses in paracrine fashion by inducing the migration of immune cells to skeletal muscles [311]. Interestingly, although PA has been reported to activate the pro-inflammatory pathway via TLR-dependent mechanisms [348], PA-induced COX-2 expression was TLR-independent. Interestingly, both OA and LA the most prevalent UFAs in the circulation [349], completely abolished PA-induced COX-2 expression as well as related PGE_2_ production via dampening of a wide array of the PA-inducible intracellular signaling processes [311].

To date, several studies have also provided supporting evidence that high plasma PA concentration induces endoplasmic reticulum (ER) stress. ER stress is a condition characterized by an accumulation of unfolded or misfolded proteins in the ER lumen, which associates with the activation of inflammatory processes and insulin resistance [312,313]. AMP-activated protein kinase (AMPK) is a crucial regulator of both cellular and whole-body energy homeostasis. Its activation has a protective effect on the development of obesity and T2DM. In this context, the suppression of AMPK has been recognized as the underlying mechanism of PA-induced ER stress, inflammation, and insulin resistance in skeletal muscle cells. In contrast, the MUFAs (OA) is capable of preventing all of these events, through AMPK-dependent mechanism [313]. Of note, the positive effect of OA on ER homeostasis has also been seen in other metabolically essential cell types, including hepatocytes and pancreatic β-cells. However, the mechanism(s) of its action in these cells remains poorly understood [350,351].

In recent years, double-stranded RNA-dependent protein kinase R (PKR), i.e., a well-known regulator of the innate immune response to viral infection, has also been recognized as the central coordinator of the functional linkage of ER stress and nutrient signals to inflammation and insulin resistance. In this regard, it has been shown that both ER stress and nutrient signals such as SFAs can stimulate PKR leading to the activation of the pro-inflammatory JNK pathway and the inhibition of insulin signaling [319]. Furthermore, PKR activity was increased in genetic and dietary murine models of obesity, especially in white adipose tissue and liver of these animals. In contrast, the lack of this enzyme prevented insulin resistance and metabolic dysfunction in obesity [319]. In general, these observations highlight the importance of the composition of fatty acids delivered to or stored within the cell as an important determinant of ER homeostasis and cellular integrity. This conclusion is not surprising as several human intervention studies point at the significance of not only the quantity but also the quality of dietary lipids in evaluating their insulin-sensitizing effects in the body [352,353].

The energy excess is one of the significant determinants of mitochondrial dysfunction, mitochondrial ROS overproduction, and insulin resistance in obese patients and rodents fed with HFD [354,355]. Moreover, mitochondrial dysfunction is currently considered as rather the consequence of hyperlipidemia-induced ROS production and insulin resistance than an early event in skeletal muscle [354,356,357]. Numerous experimental studies have provided evidence for SFAs-induced ROS overproduction in the muscle cell and the activation of stress kinases participating in the development of muscle insulin resistance. In addition, these studies also pointed at the protective effects of the monounsaturated OA against SFAs-induced damage and insulin resistance [314,315,316,317]. The exact cellular mechanisms of UFA’s actions are still unclear. However, an increased intracellular fatty acid disposal by their storage in triglycerides, as well as their oxidation in mitochondria in the case of increased energy demand, appear to be of great importance in the beneficial effects of these lipids [358]. Higher rates of mitochondrial ROS production by SFAs are reported to be involved in the activation of the NLRP3 inflammasome and weakening of insulin sensitivity, thereby supporting the existence of the relationship between HFD, inflammation, and insulin resistance [318]. In contrast, the monounsaturated OA impaired adipose NLRP3 inflammasome-mediated IL-1β secretion and insulin resistance via the preservation of AMPK activity in animal and in vitro models [359]. A recent placebo-controlled clinical trial with obese subjects, who were treated with fish oil supplements containing EPA and DHA, revealed reduced adipose tissue NRLP3 gene expression and circulating levels of IL-18, a downstream product of NLRP3 signaling [108]. Hence, these findings suggest the therapeutic potential of fish oil against metabolic diseases, such as obesity and T2DM, where the NLRP3 inflammasome has been implicated (Table 3).

The effect of PUFA dietary supplementation on glucose metabolism and diabetes control in humans has been extensively studied, but the results are inconclusive. In a systematic review and meta-analysis of 83 randomized clinical trials (RCTs) published in 2019 [320], Brown et al. concluded that increasing levels of *n*-3, *n*-6, or total PUFAs have little or no effect on prevention and treatment of T2DM. In the population of 58 643 participants with increased intake of *n*-3, there was no difference in the incidence of diabetes and no effect on glucose metabolism [320]. In addition, a RCT not included in the aforementioned meta-analysis, found no improvement in the metabolic status (including lipid control, insulin sensitivity, and adipokine profile), as well as in coagulation and inflammatory parameters in well-controlled patients with atherosclerotic vascular disease and T2DM [360]. In contrast, a 2018 meta-analysis of 45 RCTs, involving 2674 subjects with T2DM, revealed the beneficial effects of *n*-3 PUFA supplementation on lipid profile, markers of inflammation, and glycaemia in diabetic patients [361]. The discrepancies in the effectiveness of *n*-3 PUFA treatment between the studies may be, at least partially, explained by the differences in the number of participants, their clinical characteristics as well as by *n*-3 PUFAs dosage and the duration of supplementation. Because of conflicting results, no conclusions could be drawn regarding the effect of *n*-3 PUFAs on T2DM in humans. This highlights the need for highly controlled RCTs with a sufficient number of subjects and adequate duration of *n*-3 PUFA supplementation on the development and treatment of T2DM.

Taken together, in vitro and animal studies clearly indicate that UFAs have a beneficial effect on inflammatory-mediated insulin resistance in T2DM. Nevertheless, RCT data remain inconclusive, raising doubts about the clinical relevance of PUFA supplementation. Hence, more clinical trials are required to clarify their role in regulating metabolic disorders and inflammation in T2DM.

## 5. Conclusions

Dietary FAs have an influence on innate and adaptive immunity as thoroughly demonstrated in in vitro studies and animal models. Such approaches allow for in-depth mechanistic studies, usually focused on the specific cell populations. However, the clinical relevance of these results is often uncertain, due to the fact that the majority of chronic diseases in humans are multifactorial. Moreover, experimental approaches to measure the effects of FAs on immune responses often lack clarity, which leads to contrasting results. The doses used in in vitro and in vivo studies do not match physiological concentrations in biological fluids and the tissues. Furthermore, mechanistic studies, epidemiological observations, and even interventional clinical trials are heavily impacted by the lack of standardization approaches and detailed measures of all possible study confounders such as other nutritional habits, diet diversity, environmental cues, microbiome, and genetic and epigenetic heterogeneity. Finally, there are novel avenues of immunology, quickly uncovered due to technical advances in single-cell sequencing, mass spectrometry, nuclear resonance imaging, multi-omics techniques, etc., in which the effects of immunomodulation by dietary cues still need to be determined. Understanding the multidirectional effects of nutrients such as FAs on the immunological homeostasis of the organism is a must to develop personalized approaches in the prevention and treatment of many diseases.

## Figures and Tables

**Table 1:** Major effects of dietary fatty acids on innate and adaptive immune cells in vitro.

FAs	Cell Type	In Vitro Effect	Ref.
EPA	Epithelium	↓ Membrane permeability, ↑ TEER and restored TJ distribution in IECs	[31]
		↓ IL-6 and/or IL-8 production mediated by PPARγ in IECs	[34]
		↑ GPR120-dependent calcium accumulation, ↑ MAPK-ERK1/2 pathways and ↓ IL-1β-induced NF-κB activation in IECs	[36]
	Macrophages	↓ TLR4-dependent and LPS-mediated COX-2 activation and subsequent prostaglandin release	[53,54]
	Dendritic cells	↓ Activated CD1a^+^ DC frequency and ↓ GPR120 receptor expression	[55]
		↓ MHC class II (HLA-DR) and ↓ costimulatory molecule CD40, CD80, CD83, CD86 expression	[56,57,58]
		↓ Expression of IL-12p40 and TNF-α	[58]
		↓ T cell activation and ↓ IL-2 and IFN-γ production by DC-EPA-stimulated T cells	[58]
		↓ Responses to LPS, independent from NF-κB and eicosanoid system	[58]
		↓ Capacity to stimulate T cell proliferation	[34]
		↓TNF-α and IL-10 production in DCs, ↓ IFN-γ and ↑ IL-13 secretion in mixed DC-lymphocyte reaction	[34]
	Neutrophils	↑ ROS production and TNF-α production	[59]
		↓ Adhesion due to ↑ oxidized-EPA-dependent activation of PPARα	[60]
	T cells	↓ IL-2, IL-2R, TNF-α, IL-4, and IL-10 expression	[61,62,63,64,65,66]
		↓ JAK1 and JAK3 phosphorylation with the subsequent inhibition of STAT5 phosphorylation	[67]
		↓ Akt and ERK1/2 phosphorylation induced by IL-2	[67,68]
		↑ ROS-dependent cell death	[63]
	B cells	↓ IL-10, TNF-α and INF-γ production	[69]
DHA	Epithelium	↓ Membrane permeability, ↑ TEER and restored TJ distribution in IECs	[31]
		↑ Barrier functions in the presence of inflammatory conditions (IL-1β, TNF-α, IFN-γ and LPS) in IECs	[33]
		↓ IL-6 and/or IL-8 production mediated by PPARγ in IECs	[34]
		↑ GPR120-dependent calcium accumulation, ↑ MAPK-ERK1/2 pathways and ↓ IL-1β-induced NF-κB activation in IECs	[36]
		↓ IL-6 and IP-10 secretion after rhinovirus infection (AECs)	[41]
		↑ Permeability and mucus production and ↓ TEER in AECs	[43]
		↑ Healing capacity after dust extract stimulation in AECs	[42]
	Macrophages	↑ M-2 polarization regulated by p38 MAPK and mediated via PPARγ	[70,71]
		↑ GPR120-mediated M2-like polarization, ↑ phagocytic activity and ↑ IL-10 secretion	[72,73]
		↑ GRP120-dependent activation of cPLA2, ↑ release of AA, COX-2 activation and PGE_2_ release and ↑ anti-inflammatory PGE2-mediated signaling on EP4	[74]
		↓ TLR4-dependent and LPS-mediated COX-2 activation and subsequent prostaglandin release	[53,54]
	Dendritic cells	↓ Activated CD1a^+^ DC frequency and ↓ GPR120 receptor expression	[55]
		↓ MHC class II (HLA-DR) and ↓ costimulatory molecule CD40, CD80, CD83, CD86 expression	[56,57,58]
		↓ Maturation, expression of IL-12p70, IL-6, IL-23 production	[75]
		TLR2, 3, 4 and 9-dependent ↓ of IL-12p70, IL-23 and IL-27 expression mediated by PPARγ activation and ↓NF-κBp65	[56]
		↑ CD36, HLA-DR, CD83, CD86 and ↓ CD1a expression	[76]
		↓ IL-12p70 and IL-6; ↓ IL-10 expression dependent of PPARγ:RXR heterodimer	[76]
		↓TNF-α and IL-10 production in DCs, ↓ IFN-γ and ↑ IL-13 secretion in mixed DC-lymphocyte reaction	[34]
		↓ Capacity to stimulate proliferation of antigen-specific T cells and their Th1/Th17 differentiation	[34,75]
		↑ Expression of p27^Kip1^, and ↓ T-bet, GATA3, and RORγT in DC-DHA activated T cells	[75]
		↑ TGF-β and FoxP3 expression in DC-DHA activated T cells	[75]
	Neutrophils	↑ ROS production, ↑ IL-1β, and TNF-α production and ↑ phagocytosis and antifungal responses	[59]
	T cells	↓ IL-2, IL-2R, TNF-α, IL-4, and IL-10 expression	[61,62,63,64,65,66]
		↓ JAK1 and JAK3 phosphorylation with the subsequent ↓ of STAT5 phosphorylation	[67]
		↓ Akt and ERK1/2 phosphorylation induced by IL-2	[67,68]
		↓ T-cell proliferation in DCs and T cell co-culture by ↑ FoxP3^+^ T cell population	[77]
		Modulation of calcium concentration, recruiting calcium from the intracellular pool and opening calcium release-activated calcium channels	[78,79,80,81,82]
	B cells	↓ TLR4 expression, probably due to altering the membrane FA composition and the formation of the lipid rafts	[83]
		↓ IL-10, TNF-α, INF-γ and IL-6 expression	[69]
ALA	Epithelium	↓ IL-6 and/or IL-8 secretion (IECs)	[34]
	Dendritic cells	↓ Activated CD1a^+^ DC frequency and ↓ GPR120 receptor expression	[55]
LA	Epithelium	↑ Permeability and mucus production and ↓ TEER in AECs	[43]
	Dendritic cells	↑ IL-10, IL-10R and ↓ IL-12 expression, ↑ activation of ERK and NF-κB	[84]
	Neutrophils	↓ LPS-induced IL-1β secretion, mediated by ↓ TLR2, TLR4, NFKBIA, P2RX7, NLRP3, CASP-1 expression and ↑ activation of PPAR-γ after A1AT-LA stimulation	[85]
		↑ ROS production dependent from calcium signaling	[86]
	T cells	↑ ROS-induced apoptosis	[87]
Other n-6 PUFAs	Epithelium	↑ Intestinal barrier integrity after IL-4-induced inflammation (DGLA, AA) in IECs	[32]
		↓ IL-6 and/or IL-8 production mediated by PPARγ in IECs	[34]
		↑ GPR120-dependent calcium accumulation, ↑ MAPK-ERK1/2 pathways and ↓ IL-1β-induced NF-κB activation (AA) in IECs	[36]
		↑ Permeability and mucus production, ↓ TEER (AA, GLA) in AECs	[43]
		↑ Cytotoxicity and ↑ IL-6 secretion (AA) in AECs	[43,44]
		↑ IL-6 and IL-8 expression dependent on JNK and p38 MAPK signaling after AA stimulation combined with rhinovirus infection in AECs	[48]
	Dendritic cells	↓ Expression of IL-12p40 and TNF-α (AA)	[58]
		↓ T cell activation and ↓ IL-2 and IFN-γ production by EPA-DC stimulated T cells (AA)	[58]
		↓ Responses to LPS, independent from NF-κB and eicosanoid system	[58]
	Neutrophils	↑ TNFR1 and TNFR2 receptor expression mediated via PKC, ERK1/2 and cPLA_2_ (AA)	[88]
		↓ ATP production and ↑ LDH release	[89]
		↑ Mitochondria depolarization and lipid accumulation	[90]
		↑ ROS production dependent from calcium signaling (AA)	[86]
	T cells	↓ T-cell proliferation in DCs and T cell co-culture by ↑ FoxP3^+^ T cell population (AA)	[77]
OA	Neutrophils	↑ ROS production, phagocytosis and killing capacities, ↑ VEGF, CXCL3 and IL-1β production	[91,92,93]
		↑ NETosis	[94]
	T cells	↑ Apoptosis via caspase-3 pathway (high doses)	[87]
PA	Macrophages	↑ Activation of TLR4 and NF-κB pathway	[95]
		↑ IL-18 and TNF-α secretion	[96]
		↑ Activation of TLR2 and NLRP3 inflammasome and ↑ of IL-1β-secretion	[97]
	Dendritic cells	↑ TLR4 endocytosis, ↑ CD86, and CD83 expression and ↑ TLR4-dependent secretion of IL-1β via NF-κB canonical pathway	[98]
	Dendritic cells	↑ ROS production	[98]
		↑ Maturation and ↑ activation	[98]
	Neutrophils	↑ NETosis	[94]
	T cells	↑ Activation of PI3K/Akt signaling	[99,100,101]
		↑ SLAM3 expression dependent from JAK/STAT5, ↑ of TNF-α, IL-1β, IL-6, IFN-γ, IL17A and IL-2 expression	[100,102]
		↑ CD69 and ↓ CCR7 and L-selectin expression	[99]
	B cells	↓ B-cell activation (through lipoapoptosis)	[103]
Lauric acid	Dendritic cells	↑ CD40, CD80, CD86 and MHC class II expression	[104]
		↑ IL-12p70 and IL-6 secretion	[104]
		↑ TLR4 activity and T cell activation capacity	[104]
	T cells	↑ IL-2, IL-17 and GM-CS production	[105]
		↑ Th17 differentiation	[105]
		↓ Th2 differentiation	[105]
	B cells	↑ TLR4 dimerization and ↑ recruitment to lipid rafts on the plasma membrane	[83]

Table summaries studies with the observed (positive/negative) effects of dietary fatty acids on innate and adaptive immune cells in vitro. For studies showing null results, see the text. ↓—downregulation or decrease; ↑—upregulation or increase; A1AD—Alpha-1-Antitrypsin; AA—Arachidonic Acid; AECs—Airway Epithelial Cells; Akt—Protein Kinase B; ALA—Alpha-Linoleic Acid; ATP—Adenosine Triphosphate; CASP-1—Caspase-1; CCR7—C-C Chemokine Receptor Type 7; CD—Cluster of Differentiation; COX2—Cyclooxygenase-2; cPLA2—Phospholipase 2; CXCL3—Chemokine (C-X-C motif) Ligand 3; DCs—Dendritic Cells; DGLA—Dihomo-Gamma-Linolenic Acid; DHA—Docosahexaenoic Acid; EP4—Prostaglandin EP4 Receptor; EPA—Eicosapentaenoic Acid; ERK—Extracellular Signal-Regulated Kinases; FAs—Fatty Acids; FoxP3—Forkhead Box Protein 3; GLA—Gamma-Linolenic Acid; GM-CSF—Granulocyte Macrophage Colony-Stimulating Factor; GPR120—G-Protein Coupled Receptor 120; HLA-DR—Human Leukocyte Antigen—DR Isotype; IECs—Intestinal Epithelial Cells; IFN-ɣ—Interferon-Gamma; IL—Interleukin; IL-10R—Interleukin-10 Receptor; IL-2R—Interleukin-2 Receptor; JAK—Januskinase; JNK—C-Jun-N-Terminal Kinases; LA—Linoleic Acid; LDH—Lactate Dehydrogenase; LPS—Lipopolysaccharide; MAPK—Mitogen-Activated Protein Kinase; MHC II—Major Histocompatibility Complex Molecules Class II; NETosis—Neutrophil Extracellular Traps Formation; NF-kB—Nuclear Factor Kappa-Light-Chain-Enhanced of Activated B cells; NFKBIA—NFKB Inhibitor Alpha; NLRP3—NOD-like Receptor Protein 3; p27^Kip1^—Cyclin-Dependent Kinase Inhibitor 1B; P2RX7—Purinergic Receptor P2X 7; p38 MAPK—p38 Mitogen-Activated Protein Kinases; PGE2—Prostaglandin E2; PI3K—Phosphoinositide 3-Kinase; PKC—Protein Kinase C; PPARɣ—Peroxisome Proliferator-Activated Receptor Gamma; PPARα—Peroxisome Proliferator-Activated Receptor Alpha; PUFAs—Polyunsaturated Fatty Acids; RORɣT—RAR-Related Orphan Receptor Gamma; ROS—Reactive Oxygen Species; RXR—Retinoid X Receptor; SLAM3—Signaling Lymphocytic Activation Molecule 3; STAT5—Signal Transducer and Activator of Transcription 5; T-bet—T-cell Associated Transcription Factor; TEER—Trans Epithelial Electrical Resistance; TGF-β—Tumor Growth Factor-Beta; Th1—Type 1 T Helper Cells; Th17—Type 17 T Helper Cells; Th2—Type 2 T Helper Cells; TJs—Tight Junctions; TLR—Toll-like Receptor; TNF-α—Tumor Necrosis Factor-Alpha; TNFR1—Tumor Necrosis Factor Receptor 1; TNFR2—Tumor Necrosis Factor Receptor 2; VEGF—Vascular Endothelial Growth Factor; ZO-1—Zonula Occludens-1.

**Table 2:** Major effects of *n*-3 PUFA- and SFA-enriched diets on innate and adaptive immune cells in animal models and clinical trials.

Diet	Cell Type	In Vivo Effect	Ref.
*n*-3 PUFA-rich diet	Epithelium	↑ Barrier function in the mouse model of intestinal anaphylactic response	[37]
		↓ Inflammatory score, ↑ TJ proteins expression (ZO-1 and Occludin), ↓ IL-17, TNF-α and IFN-γ production in the IL-10-deficient model of chronic colitis	[38]
		↓ Intestinal pathology scores and IL-12, TNF-α, IL-1β secretion and ↑ of ZO-1 expression in SCID mice in the model of colitis	[39]
		↓ Oxidative stress (8-IP, glutathione and iNOS) in a rat model of colitis	[40]
		↑ Healing capacity during dust-stimulated lung inflammation	[42]
	Macrophages	↓ Frequency of ATMs and ↑ M2 anti-inflammatory phenotype and ↑ expression of IL-10, arginase, YM-1, Clec7a and MMR	[72]
		↑ Phagocytosis, microbicidal activity and ↓ apoptosis	[106,107]
		↓ NLRP3 inflammasome activation via GPR120 and GPR40 receptors and their downstream protein β-arrestin-2	[108,109]
		↑ Autophagy	[110]
	Dendritic cells	↓ Capacity to stimulate proliferation of antigen-specific T cells and their Th1/Th17 differentiation	[75]
		↓ Antigen-presenting properties and ↓ CD2^+^ DCs, and ↓ CD18, CD11a, HLA-DR, CD54 expression	[111]
		↓ CD80 and CD11c^+^ expression, ↓ TNF-α production and ↓ phagocytosis	[112]
		↓ Th1-inducing, pro-inflammatory (CD11c^+^ CD11b^−^ CD8α^+^) lymphoid DCs and ↑ myeloid, tolerogenic (CD11c^+^ CD11b^+^ CD8α^−^) DCs subpopulations	[112,113]
		↓ T cell proliferation and ↓ IFN-γ and IL-17 production in the DC-lymphocyte reaction	[114]
	Neutrophils	Protection during *S. aureus* sepsis, with ↓ mortality and bacteria load	[115]
		↑ CD11b^high^, Ly6G^high^ and MHC class II^high^ neutrophil subpopulation in the blood	[116]
		*↑ Specialized pro-resolving mediators, such as 18-HEPE, RvE1, RvE2, RvE3, 17-HDHA and RvE5*	[117]
		*↑ Neutrophil telomere length, probably due to ↓ oxidative stress*	[118]
		*↑ Membrane integrity, ↓ ROS production, and mitochondrial membrane potential after exercise*	[119]
		*↑ Functions and frequency in oncologic patients undergoing chemotherapy*	[120]
		↑ Neutrophil-dependent inflammation in mice genetically susceptible to colitis	[121]
	T cells	↓ Proliferation via ↓ IL-2 secretion and IL-2RA expression	[122]
		↓ Production of diacylglycerol, ceramide, and level of phospholipase Cγ	[122]
		Disrupted spatial organization of the second messenger, PI(4,5)P2, perturbing downstream signals required for T cell proliferation	[123]
		↓ Frequency of pro-inflammatory T cells in the fat tissues, mediated by CCR-4, CXCR4 and ↓ expression of P-selectin and ICAM-1 on the endothelium	[66,124]
		↓ Formation of pseudopods and ↓ ratio between F-actin and G-actin and ↓ Rhoα and Rac1 involved in cell migration	[125]
		↓ IL-6, IL-23, IL-17 expression and ↑ FoxP3, CTLA-4, TGF-β and IL-10 expression	[66,126,127]
		↓ Treg-dependent proliferation of T effector cells mediated by ↓ of ERK1/2 and Akt phosphorylation and ↑ histone deacetylase and p27^Kip1^ expression	[66]
	B cells	↑ CD69 and CD40 expression	[103,128]
		↑ IL-6, IFN-γ, TNF-α and IL-10, IL-5, IL-13, and IL-9 expression	[103,128,129]
		↑ Percentage of splenic transitional, marginal zone B cells and peritoneal B1 cells	[130,131]
		↑ Surface expression of IgM in spleen and serum	[130,131]
		↑ Caecal IgA	[128]
		Changed lipid composition of B cells and ↑ size, order and the distribution of rafts	[128,129,132]
SFA-rich diet	Dendritic cells	↓ Tolerogenic (CD11c^+^ CD103^+^ CD11b^+^) and ↑ pro-inflammatory (CD11c^+^ CD103^−^ CD11b^+^) subpopulations in the gut	[133]
		↑ TLR4-dependent NLRP3 inflammasome activation and IL-1β secretion	[134]
	Neutrophils	↓ Survival rate and ↑ bacterial proliferation in septic mice	[135]
		↓ Cell frequency, ↓ phagocytosis and ↓ ROS production in septic mice	[135]
	T cells	↑ CXCR3 and ↓ CCR7 and L-selectin expression	[99]
		↑ Severity of the disease, ↑ Th1, and Th17 cell differentiation and ↑ T cell infiltration into the central nervous system	[101,136]
		*Positive association with several markers of inflammation, such as C-reactive protein, IL-1RA, IFN-γ and CCL5*	[137]

Table summaries studies with the observed (positive/negative) effects of *n*-3 PUFA- and SFA-enriched diets on innate and adaptive immune cells in animal models and clinical trials. For studies showing null results, see the text. ↓—downregulation or decrease; ↑—upregulation or increase; 17-HDHA—17-Hydroxydocosahexaenoic Acid; 18-HEPE—18-Hydroxyeicosatetraenoic Acid; 8-IP—8-Isoprostane; Akt—Protein Kinase B; ATMs—Adipose Tissue Macrophages; CCL5—C-C Chemokine Ligand 5; CCR-4—C-C Chemokine Receptor Type 4; CCR7—C-C Chemokine Receptor 7; CD—Cluster of Differentiation; CD11b—Integrin Alpha M; Clec7a—C-type Lectin Domain Family 7 Member A; CTLA-4—Cytotoxic T-Lymphocyte-Associated Protein 4; CXCR3—C-X-C Chemokine Receptor Type 3; CXCR4—C-X-C Chemokine Receptor Type 4; DCs—Dendritic Cells; ERK—Extracellular Signal-Regulated Kinases; FoxP3—Forkhead Box Protein 3; GPR120—G-Protein Coupled Receptor 120; GPR40—G-Protein Coupled Receptor 40; HLA-DR—Human Leukocyte Antigen—DR Isotype; ICAM-1—Intercellular Adhesion Molecule 1; IFN-ɣ—Interferon-Gamma; IgA—Immunoglobulin A; IgM—Immunoglobulin M; IL—Interleukin; IL-1RA—Interleukin 1 Receptor Antagonist; IL-2RA—Interleukin 2 Receptor Alpha Chain; iNOS—Nitric Oxide Synthase; Ly6G—Lymphocyte Antigen 6 Complex Locus G6D; MHC class II—Major Histocompatibility Complex Molecules Class II; MMR—Macrophage Mannose Receptor; NLRP3—NOD-like Receptor Protein 3; p27^Kip1^—Cyclin-Dependent Kinase Inhibitor 1B; PI(4,5)P2—Phosphatidylinositol-4,5-Bisphosphate; PUFAs—Polyunsaturated Fatty Acids; ROS—Reactive Oxygen Species; RvE—Resolvin E; SCID—Severe Combined Immunodeficiency; SFAs—Saturated Fatty Acids; TGF-β—Tumor Growth Factor Beta; Th1—Type 1 T helper Cells; Th17—Type 17 T helper Cells; TJs—Tight Junctions; TLR—Toll-like Receptor; TNF-α—Tumor Necrosis Factor-Alpha; Treg—T Regulatory Cells; ZO-1—Zonula Occludens 1. Clinical trials are presented in italics.

**Table 3:** Major effects of a diet enriched with fatty acids on allergic, autoimmune, and metabolic diseases.

**Allergic Diseases**			**Effects of n-3 PUFAs**		**Ref.**
Asthma	Pregnancy and lactation	Prevention of asthma and allergic disease development in the offspring			[136,196,214,216,217]
		↑ Risk of allergic diseases and ↓ lung function in the offspring associated with high *n*-3 PUFAs in the colostrum			[249]
	Infancy and childhood	↓ Risk of asthma, rhinitis and aeroallergen sensitization			[200,204,220,221]
		↓ Asthma symptoms scores and ↓ responsiveness to acetylcholine			[222]
		Improvement in pulmonary functions and ↓ use of short-acting inhaled bronchodilators and inhaled corticosteroids			[290]
		↑ Prevalence of wheeze			[248]
	Adulthood	↓ Prevalence of asthma symptoms, airway hyperresponsiveness and ↑ asthma control and lung function			[47,202,206,207,224,226,227,228,230,233]
		↓ Exhaled nitric oxide, ↓ serum eosinophils and ↑ FEV1			[225,229]
		↓ Bronchodilator use, ↓ exhale nitric oxide and ↓ severity of exercise-induced bronchoconstriction			[228,231,232,233]
Allergic Rhinitis	Infancy and childhood	↓ Risk of rhinitis development			[251,254,255,256]
	Adulthood	↓ Prevalence			[257,258]
Atopic Dermatitis	Pregnancy and lactation	↓ Incidence and intensity in the offspring			[259,260]
	Infancy and childhood	↓ Frequency of eczema			[261]
	Adulthood	↓ Severity and symptoms			[271,272,275]
		↑ SCORing Atopic Dermatitis (SCORAD) after 4, 8 and 16 weeks of treatment			[273]
Food Allergy	Pregnancy and lactation	↓ Allergic sensitization to food proteins (antigen-dependent effect)			[196,260,277,278,279,280,289]
	Infancy and childhood	↓ Odds ratio for food allergy and ↓ risk for sensitization (antigen-dependent effect)			[259,277,281,282,287]
**Autoimmune Diseases**		**Effects of *n*-3 PUFAs**			**Ref.**
Rheumatoid Arthritis		Positive correlation with synovial fluid composition and inversed correlation with pain score			[291]
		↓ Incidence and improved prognosis			[292,293,294,295,296]
		↓ Duration of morning stiffness and ↓ number of tender and swollen joints			[297]
Polymyositis and Dermatomyositis		↑ Skeletal muscle growth and functions			[298]
Sjögren Syndrome		↓ Inflammation and ↑ barrier function in the salivary glands			[299,300]
Systemic Lupus Erythematosus		↓ Inflammatory mediators, leukocyte chemotaxis and adhesion molecules expression			[301]
Antiphospholipid Syndrome		↑ Endothelial function			[302]
Multiple Sclerosis		↑ M2-like macrophages shift, prevented de-myelinization, promoted neuroprotection and re-myelinization ↓ incidence			[303,304,305,306]
Type 1 Diabetes		Protection against T1DM in infants, no protective effect in older children reported			[307,308]
**Metabolic Diseases**		**Effects of FAs**			**Ref.**
Type 2 Diabetes		SFAs		↑ Intramyocellular accumulation of DAG and/or ceramide, ↑ PKCθ activation and ↑ NF-κB-dependent expression of IL-6 and TNF-α	[309,310]
				↑ TLR-independent PGE2 production via COX-2 and p38 MAPK pathways	[311]
				↓ AMPK activation, with subsequent ↑ ER stress associated with inflammation and insulin resistance	[312,313]
				↑ ROS production in muscle cells and muscle insulin resistance development	[314,315,316,317]
				↑ ROS production and ROS-dependent ↑ NLRP3-inflammasome activation and insulin resistance	[318]
				↑ PKR activation and induction of JNK inflammatory pathways and inhibition of insulin signaling	[319]
		UFAs		↓ PA-dependent COX-2 expression and related PGE2 production (OA, LA)	[311]
				↓ PA-dependent ER stress associated with reduced inflammation and insulin resistance (OA)	[313]
				↓ PA-dependent muscle destruction and insulin resistance development (OA)	[317]
				↓ NLRP3-inflammasome-dependent IL-1β secretion in adipose tissue and insulin resistance (EPA, DHA)	[108]
				Limited or no effect of PUFA supplementation on T2DM prevention and treatment	[320]

Table summaries studies with the observed (positive/negative) effects of diet enriched with dietary fatty acids on allergic, autoimmune and metabolic diseases. For studies showing null results, see the text. ↓—downregulation or decrease; ↑—upregulation or increase; AMPK—AMP-Activated Protein Kinase; COX-2—Cyclooxygenase-2; DAG—Diacylglycerol; DHA—Docosahexaenoic Acid; EPA—Eicosapentaenoic Acid; ER—Endoplasmic Reticulum; FAs—Fatty Acids; FEV1—Forced Expiratory Pressure in 1 Second; IL—Interleukin; JNK—C-Jun-N-Terminal Kinases; LA—Linoleic Acid; NF-kB—Nuclear Factor Kappa-Light-Chain-Enhanced of Activated B Cells; NLRP3—NOD-like Receptor Protein 3; OA—Oleic Acid; p38 MAPK—p38 Mitogen-Activated Protein Kinases; PA—Palmitic Acid; PGE2—Prostaglandin E2; PKCθ—Protein Kinase C-Theta; PKR—Protein Kinase R; PUFAs—Polyunsaturated Fatty Acids; ROS—Reactive Oxygen Species; SFAs—Saturated Fatty Acids; T1DM—Type 1 Diabetes Mellitus; T2DM—Type 2 Diabetes Mellitus; TLR—Toll-like Receptor; TNF-α—Tumor Necrosis Factor-Alpha; UFAs—Unsaturated Fatty Acids.

## Abbreviations

AA	Arachidonic Acid
Akt	Protein Kinase B
ALA	α-Linolenic Acid
BM-DCs	Bone Marrow-Derived Dendritic Cells
CD	Cluster of Differentiation
COX	Cyclooxygenase
DCs	Dendritic Cells
DHA	Docosahexaenoic Acid
EAE	Experimental Autoimmune Encephalomyelitis
EPA	Eicosapentaenoic Acid
ERK	Extracellular Signal-Regulated Kinase
FAs	Fatty Acids
GLA	γ-Linolenic Acid
GPRs	G-Protein-Coupled Receptors
HFD	High Fat Diet
IBD	Inflammatory Bowel Disease
IFN	Interferon
IL	Interleukin
ILCs	Innate Lymphoid Cells
JAK	Janus Kinases
JNK	C-Jun N-Terminal Kinase
LA	Linoleic Acid
LPS	Lipopolysaccharide
MA	Myristic Acid
MAPK	Mitogen-Activated Protein Kinase
mo-DCs	Monocyte-Derived Dendritic Cells
MUFAs	Monounsaturated Fatty Acids
NLRP3	NOD-like Receptor Protein 3
OA	Oleic Acid
PA	Palmitic Acid
PGE2	Prostaglandin E_2_
PKC	Protein Kinase C
PPAR	Peroxisome Proliferator-Activated Receptor
PUFAs	Polyunsaturated Fatty Acids
ROS	Reactive Oxygen Species
SFAs	Saturated Fatty Acids
STA	Stearic Acid
STAT	Signal Transducer and Activator of Transcription Proteins
TEER	Trans Epithelial Electrical Resistance
TJs	Tight Junctions
TLRs	Toll-Like Receptors
UFAs	Unsaturated Fatty Acids
